# TGF-β Regulation of T Cells

**DOI:** 10.1146/annurev-immunol-101921-045939

**Published:** 2023-02-07

**Authors:** WanJun Chen

**Affiliations:** Mucosal Immunology Section, National Institute of Dental and Craniofacial Research, National Institutes of Health, Bethesda, Maryland, USA

**Keywords:** CD4^+^ T helper cells, CD8^+^ T cells, regulatory T cells, innate lymphoid cells, intraepithelial lymphocytes, *γ*δ T cells, T cell quiescence and activation

## Abstract

Transforming growth factor β (TGF-β) is a key cytokine regulating the development, activation, proliferation, differentiation, and death of T cells. In CD4^+^ T cells, TGF-β maintains the quiescence and controls the activation of naive T cells. While inhibiting the differentiation and function of Th1 and Th2 cells, TGF-β promotes the differentiation of Th17 and Th9 cells. TGF-β is required for the induction of Foxp3 in naive T cells and the development of regulatory T cells. TGF-β is crucial in the differentiation of tissue-resident memory CD8^+^ T cells and their retention in the tissue, whereas it suppresses effector T cell function. In addition, TGF-β also regulates the generation or function of natural killer T cells, *γ*δ T cells, innate lymphoid cells, and gut intraepithelial lymphocytes. Here I highlight the major findings and recent advances in our understanding of TGF-β regulation of T cells and provide a personal perspective of the field.

If something in the cell is “off,” TGF-β may turn it “on,” while if something in the cell is “on,” TGF-β may turn it “off.”

—M.B. Sporn (1, p. 6)

## 1. INTRODUCTION

The delicate balance between effective immunity and proper tolerance is the prerequisite for an unerring immune system and for the well-being of the human body. Among the numerous immune cells, T cells are a key population in adaptive immunity. Developed in the thymus, T cell receptor (TCR)αβ^+^CD4^+^ and TCRαβ^+^CD8^+^ T cells and TCRγδ^+^ T cells migrate into the blood and peripheral lymphoid tissues and maintain their quiescence as naive T cells under intrinsic and extrinsic regulatory forces until they encounter their specific antigens processed and presented by antigen-presenting cells (APCs). Upon TCR stimulation, naive T cells can be activated with the assistance of positive costimulatory molecules such as CD28 and simultaneously by suppression and/or removal of negative factors. T cells can then produce IL-2, proliferate, and differentiate into effector cells, memory cells, or regulatory cells depending on the cytokines and molecules present in their microenvironments. Based on their function, cytokine production, and expression of lineage transcription factors, CD4^+^ T cells can be classified as T helper 1 (Th1), Th2, Th17, Th9, and T follicular helper (Tfh) effector cells; forkhead box P3 (Foxp3)^+^ regulatory T cells (Tregs); and T follicular regulatory T (Tfr) cells (2–9). On the other hand, CD8^+^ T cells can be divided into effector (Teff ), exhausted (Tex), central memory (Tcm), effector memory (Tem), and resident memory (Trm) subpopulations, as well as CD8^+^ Tregs (10, 11). In addition, several nonconventional lymphocytes have been discovered and found to play important roles in immune responses. These include but are not limited to innate lymphoid cells (ILCs) (12), intraepithelial lymphocytes (IELs) in the gut (13), and natural killer T (NKT) cells (14). The development, activation, proliferation, differentiation, and death of T cells require extremely orchestrated signals, molecules, and cytokines no matter whether TCR engagement is needed or not. TGF-β is one of the most important cytokines in the regulation of T cells.

TGF-β was discovered in mouse tissues by Roberts, Sporn, and colleagues in 1981 (15). It is now known that the TGF-β superfamily is composed of at least 33 members including TGF-β, bone morphogenetic proteins (BMPs), activins, inhibins, and glial cell line–derived neurotrophic factors (GDNFs) (16). TGF-β has three mammalian isoforms: TGF-β1, TGF-β2, and TGF-β3 (TGF-β1, 2, 3). Although TGF-β1, 2, 3 utilize the same receptors and reactive Smad2 and Smad3 (RSmad) signaling in vitro, the different isoforms may function differently in vivo. For example, TGF-β2 is essential in controlling the development of multiple vital organs in the body, as null mutation of the *Tgfb2* gene results in a lethal embryo (17). TGF-β3 also controls the development of some organs such as lungs and accelerates wound healing without an increase in scar formation (18, 19). TGF-β1, however, is the dominant isoform in blood and immune cells and plays the most important roles in regulating immune responses, as null mutation of the *Tgfb1* gene (*Tgfb1*^−/−^) causes systemic inflammation and untimely death in mice (20, 21). Importantly, specific deletion of TGF-β receptor I (TGFβRI) or TGFβRII or Smad2/3 in T cells leads to systemic T cell activation, uncontrolled inflammation, and early demise of all mice, similar to what is seen in *Tgfb1*^−/−^ mice (22–25). I use TGF-β to represent TGF-β1 in this review unless otherwise specified.

**Figure 1:**
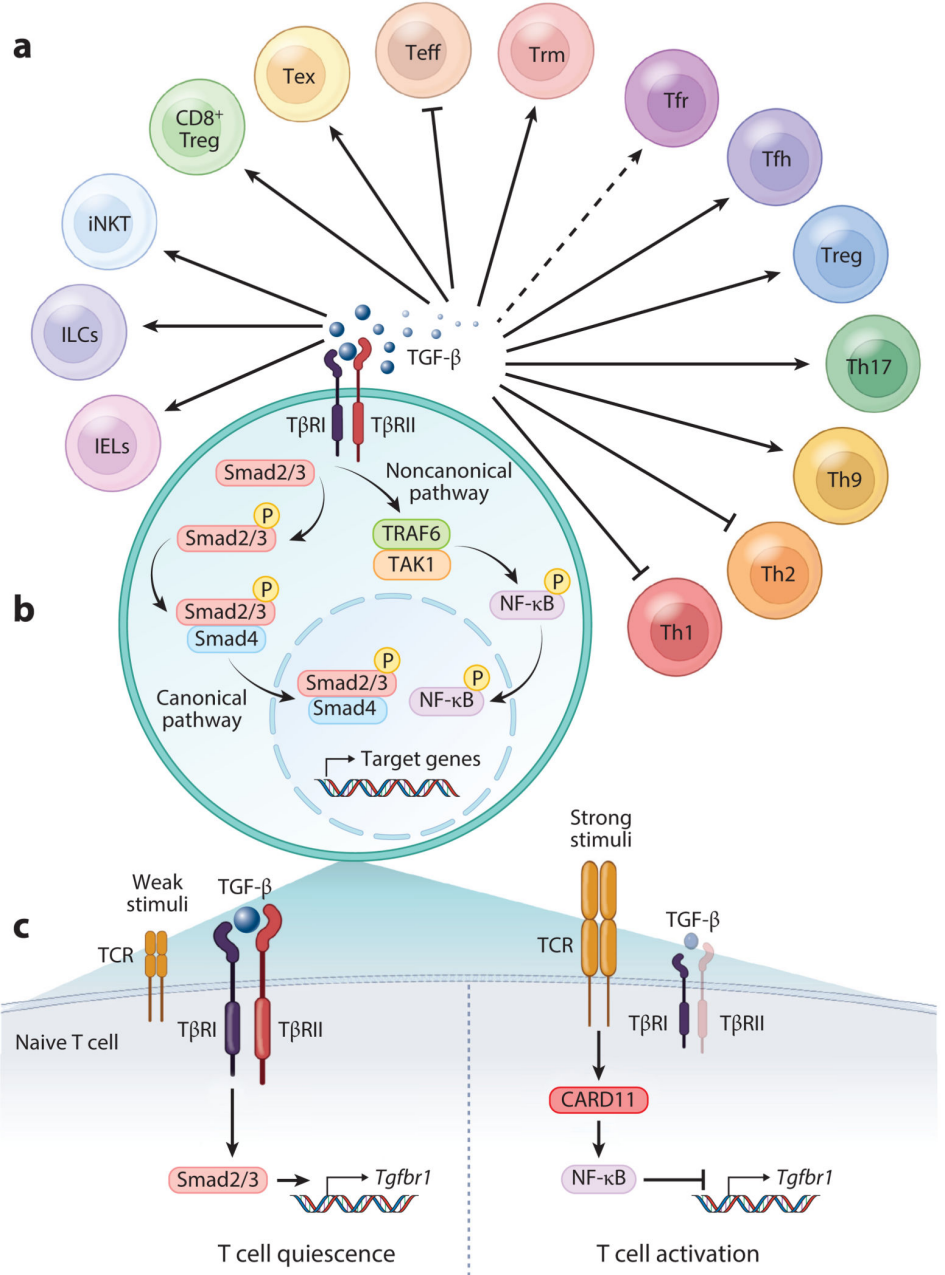
TGF-β regulation of T cells. (*a*) In CD4^+^ T cells: Th1, Th2, Th9, and Th17 cells; CD25^+^Foxp3^+^ Tregs, including thymic Tregs and induced/peripheral Tregs; Tfh cells; and Tfr cells. In CD8^+^ T cells: Trm cells, Teff cells, Tex cells, and CD122^+^Ly49^+^Foxp3^−^ Tregs. In addition, iNKT cells, ILCs, and gut IELs. Dashed arrow indicates no experimental evidence available. (*b*) General TGF-β signaling pathways. TGF-β engages TβRII and then recruits and phosphorylates TβRI. The receptor complex then initiates the canonical Smad2- and Smad3-dependent pathway and/or noncanonical pathways such as the TRAF6-TAK1-dependent pathway to regulate target gene expression. (*c*) TCR-mediated regulation of TGF-β-TβRI signaling acts as a third signal in determining T cell quiescence and activation. Naive CD4^+^ T cells in mice and humans have active TGF-β signaling, evidenced by TβRI, TβRII, and activated Smad2/3 (pSmad2/3). TGF-β signaling represents a key intrinsic negative signal in retaining quiescence and preventing unwanted activation in naive CD4^+^ T cells. After TCR engagement, naive CD4^+^ T cells show no reduction of, and very likely higher, TβRI expression in response to weak or low-dose TCR stimuli (*left*), but they exhibit rapid and profound reduction of TβRI when activated with strong or high-dose TCR stimuli in a CARD11-NF-κB-dependent manner (*right*). Data not represented here indicate that TGF-β preserves and even upregulates the TCR-mediated downregulation of TβRI and that IL-6 abolishes TGF-β-mediated upregulation of TβRI in the context of strong TCR stimulation. Abbreviations: CARD11, caspase recruitment domain–containing protein 11; IEL, intraepithelial lymphocyte; ILC, innate lymphoid cell; iNKT, invariant natural killer T; pSmad2/3, phosphorylated Smad2/3; TAK1, TGF-β-activated kinase 1; TβRI, TGF-β receptor I; TCR, T cell receptor; Teff, effector T; Tex, exhausted T; Tfh, follicular helper T; Tfr, follicular regulatory T; Th1, T helper type 1; TRAF6, TNF receptor–associated factor 6; Treg, regulatory T cell; Trm, T resident memory. Figure adapted from images created with BioRender.com.

TGF-β is produced by and responds to almost all immune cells and nonimmune cells and regulates both innate and adaptive immune responses. There have been numerous excellent reviews of TGF-β biology, including its functions in cell biology, cancer biology, and immune responses (16, 26–31). In this review, I focus on TGF-β regulation of T cells (Figure 1). I briefly summarize the general TGF-β signaling pathway and then discuss the major findings and recent advances in research on the cellular and molecular mechanisms of TGF-β regulation of T cells in physiological and pathological conditions including autoimmunity, cancer, and infectious diseases.

## 2. TGF-β SIGNALING

TGF-β is produced as a biologically inactive precursor protein and requires proteolytic processing. The latent form of TGF-β [LAP–TGF-β (LTGF-β)] contains a large N-terminal portion known as the latency-associated polypeptide (LAP) and a noncovalently associated C-terminal peptide, the active TGF-β. In some cases, LTGF-β can be chemically linked by disulfide bonds with two cysteine residues to form so-called latent TGF-β binding proteins (LTBPs) that can interact with and bind to proteins in the extracellular matrix (31). LTGF-β can be activated by removing the LAP portion from the precursors to release the bioactive TGF-β. Numerous proteinases and proteins in the matrix, such as matrix metalloproteinases (MMPs), and cell membrane–bound integrins, such as α_v_β_8_ in Tregs and dendritic cells (DCs) and α_v_β_6_ in epithelial cells, have been implicated in the process of TGF-β activation (31–33). T cell–conditional deletion of furin, which is involved in TGF-β activation, causes activation and proliferation of Teff cells, which produce less bioactive TGF-β1 (34). It is conceivable that different factors and processes are required for TGF-β activation in different tissues and different immunological conditions, but the amounts of available bioactive TGF-β may determine the outcome for even the same T cells encountering the same antigen stimulation.

Once activated, TGF-β signals by binding to specific sets of heteromeric type I and type II receptor complexes (16, 26, 29). TGF-β first binds to TβRII and then recruits and activates TβRI (also called ALK5). In the canonical pathway, activated TβRI recruits and phosphorylates the receptor RSmad. Smad2/3 can be phosphorylated at the C terminus or in the linker region in response to TGF-β in the presence or absence of other signals. Phosphorylated Smad2/3 (pSmad2/3) normally forms a complex with the common Smad (Smad4) to be translocated into the nucleus to regulate their target genes. In general, the Smad2/3/4 complex is weak or even unable to regulate target gene transcription without interacting and/or collaborating with other cotranscription factors. Smad3 and Smad2 can compensate for each other in mediating TGF-β signaling in immune cells, as deletion of both Smad2 and Smad3, but not either one alone, in T cells results in complete block of TGF-β signaling (25). In addition to the canonical RSmad-mediated pathway, TGF-β can also signal through a TGF-β receptor–mediated but RSmad-independent noncanonical pathways (26). For example, the TNF receptor–associated factor 6 (TRAF6)-TGF-β-activated kinase 1 (TAK1) axis can serve as a critical route downstream of TGF-β receptors to mediate TGF-β signaling (35, 36) (Figure 1). Of special note, when considering TGF-β signaling in immune cells, especially in T cells, one must keep in mind that the presence of other stimuli and signals may determine and/or change the ultimate fate of the T cells.

## 3. TGF-β IN THYMOCYTES

Although compelling evidence indicates that TGF-β controls the development of thymic Foxp3^+^ Tregs (23, 37–40), invariant NKT (iNKT) cells (24), and precursors of TCRαβ^+^CD8αα^+^ IELs (41) in the thymus, the function of TGF-β regulation of normal thymocyte development remains largely unclear (see Supplementary Text 1), with the exception of CD8 single-positive (SP) thymocytes. It has been reported that TGF-β is required for the development of CD8 SP thymocytes. An elegant study by Singer’s group has clearly demonstrated that TGF-β is indispensable in CD8 SP lineage decision (42). The authors revealed that four cytokines, namely IL-6, IFN-γ, TSLP (thymic stromal lymphopoietin), and TGF-β, together induce expression of the lineage-specifying transcription factor Runx3d (runt-related transcription factor 3d) and signal the generation of CD8^+^ T cells in the thymus. They conclude that it is a combination of signals (*a*) by IL-7 and IL-15 through common γ chain cytokine receptor; (*b*) by IL-6, IFN-γ, and TSLP through Jak-Stat; and (*c*) by TGF-β through Smad3 that completes the generation of CD8^+^ SP thymocytes (42). Consistent with this finding, it is reported that TGF-β upregulates IL-7 receptor expression in CD8^+^ SP thymocytes (43) (Supplementary Figure 1).

## 4. TGF-β IN CD4^+^ T CELLS

TGF-β regulates the quiescence, activation, proliferation, differentiation, and death of CD4^+^ T cells. The delicate balance of TGF-β regulation in all the aforementioned processes safeguards the normal function of CD4^+^ T cells.

### 4.1. TGF-β in CD4^+^ T Cell Quiescence and Activation

A fundamental question in immunology is how quiescence is enforced in naive T cells while activation by foreign antigens and self-antigens is allowed. After leaving the thymus, naive CD4^+^ T cells are present in the periphery in quiescence and survive in the steady sate, which requires TCR tickling by self-MHC molecules (44). However, TCR tickling by self-antigens does not lead to autoimmunity in healthy individuals, as T cell quiescence is actively reinforced by extrinsic factors such as Tregs and potentially by intrinsic mechanisms. Naive CD4^+^ T cell activation requires two signals, the first being the signal of TCR engagement and the second being provided by costimulatory molecule CD28 (45). It is conceivable that in addition to these positive signals, the putative negative signal(s) from quiescence programs must be removed to achieve T cell activation. Indeed, Tu et al. (46) revealed that active TGF-β signaling occurs in both murine and human naive CD4^+^ T cells and strong, not weak, TCR engagement reduces TGF-β signaling by downregulating TβRI and consequent pSmad2/3 in CD4^+^ T cells. This TβRI downregulation occurs as early as 6–12 h after TCR stimulation and occurs through activation of CARD11 (caspase recruitment domain–containing protein 11) and NF-κB. In examining which cytokines influence TCR-mediated TβRI downregulation, we found that of the panel of immune cytokines tested, only TGF-β prevents TCR-mediated TβRI downregulation. Interestingly, IL-6 is able to abolish TGF-β-mediated upregulation of TβRI, although IL-6 itself has no effects on TCR-mediated TβRI downregulation in CD4^+^ T cells (46). Functional analysis reveals that indeed, downregulation of TβRI and pSmad2/3 increases the sensitivity of T cell proliferation and production of cytokines such as IFN-γ in response to TCR stimulation, especially weaker TCR stimulation. Conversely, overexpression of TβRI in naive and activated T cells rendered T cells less responsive to TCR stimulation and suppressed autoimmunity (46). Significantly, naive CD4^+^ T cells isolated in newly diagnosed and untreated patients with systemic lupus erythematosus manifested reduced TβRI expression and increased TCR-driven proliferation compared to cells from healthy subjects (46). However, more studies are needed to validate these findings. Although these findings establish a key role for TGF-β signaling in maintaining the quiescence and controlling the activation of naive CD4^+^ T cells, the molecular pathways downstream of TGF-β-Smad2/3 signaling remain unknown. Nonetheless, these findings indicate that TCR-mediated regulation of TGFβ-TβRI signaling is a crucial criterion in determining T cell quiescence and activation (Figure 1), which should have important implications for our understanding of immune tolerance and immune responses, as well as the development and pathogenesis of autoimmunity, cancer, and infectious diseases (Supplementary Text 2).

### 4.2. TGF-β in CD4^+^ T Cell Differentiation and Function

**Figure 2:**
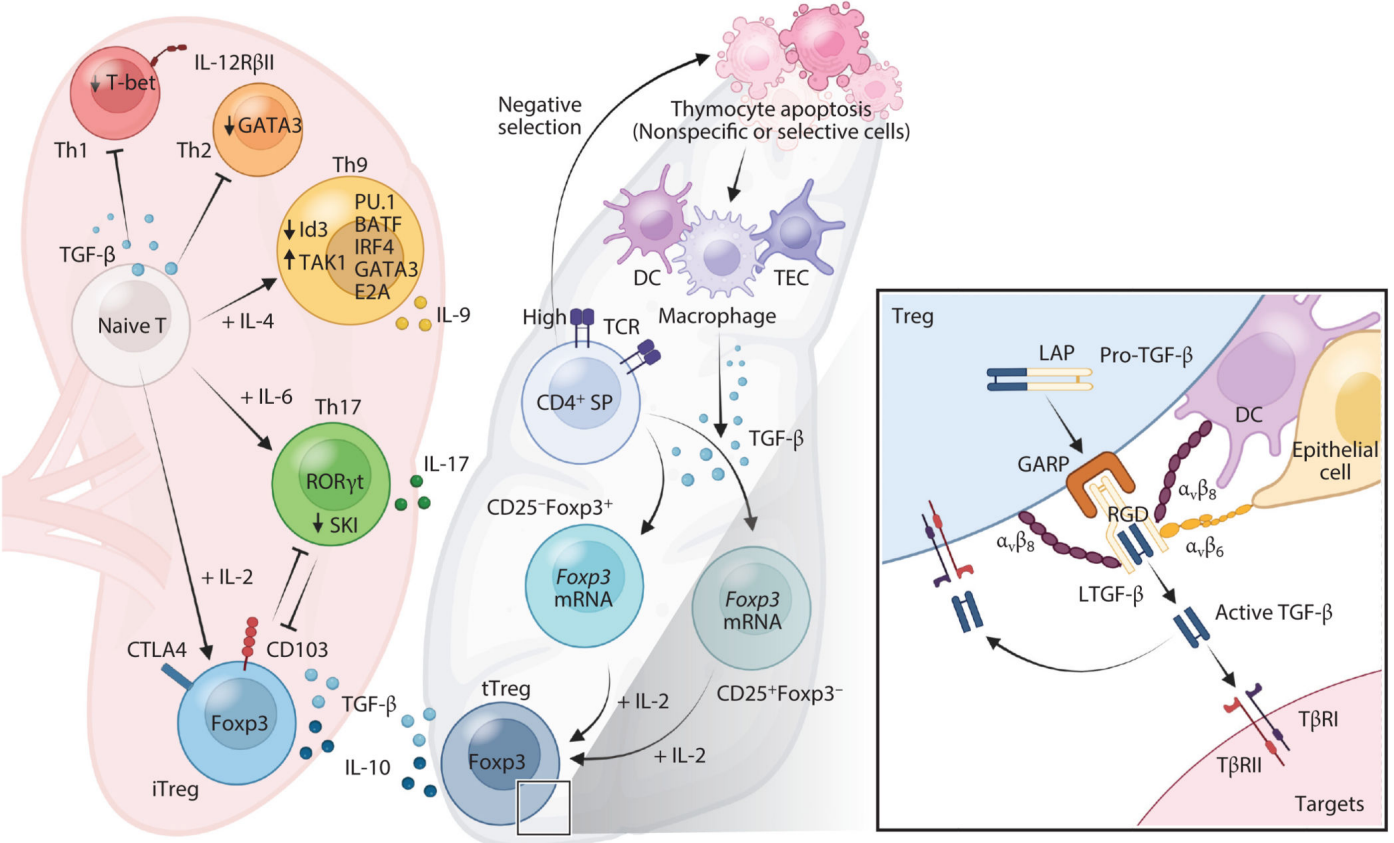
(*Left*) TGF-β regulation of CD4^+^ T cell differentiation and function. TGF-β suppresses T-bet and IL-12RβII in Th1 cells, and GATA3 in Th2 cells. Multiple transcription factors, including PU.1, BATF, IRF4, GATA3, TAK1, ID3, and E2A, have been reported to be involved in regulation of *Il9* gene activation during Th9 differentiation in response to TGF-β and IL-4. For example, TGF-β suppresses Id3 expression through a TAK1-dependent pathway, and this process enhances the activity of E2A and GATA3 in *Il9* gene transcription. During Th17 differentiation, TGF-β induces RORγt expression, which can be enhanced by IL-6. Smad2/3 are required for Th17 differentiation, although it is still controversial whether Smad2/3 are required for TGF-β induction of RORγ. In contrast, Smad4 inhibits Th17 differentiation by directly interacting with SKI, and TGF-β disrupts SKI and reverses Smad4-SKI complex–mediated inhibition of RORγt. TGF-β is essential for induction of Foxp3 in naive CD4^+^ T cells and converts them into Foxp3^+^ Tregs (iTregs/pTregs). (*Middle*) TGF-β is required for the development of tTregs. A proposed model of thymocyte apoptosis linked to the generation of tTregs in a TGF-β-dependent manner. Thymocytes undergoing apoptosis (e.g., negative selection) can be taken up and digested by macrophages, DCs, and thymic epithelial cells in the thymus, and this results in TGF-β production by these phagocytes and/or activation by yet unidentified mechanisms. TGF-β in turn induces the *foxp3* gene from Foxp3^−^CD4^+^CD8^−^ single-positive cells that simultaneously are engaged by their specific antigenic peptides (e.g., self-antigens). This results in two lineage-committed thymic Treg precursors (or immature thymic Tregs): CD25^+^Foxp3^−^ ( *foxp3* mRNA^+^) and CD25^−^Foxp3^+^ precursors. These two precursors can then mature into CD4^+^CD25^+^Foxp3^+^ thymic Tregs in response to IL-2 in the thymus. This model could reconcile with the current models of thymic Treg development (Supplementary Text 6). (*Right*) TGF-β in Treg function. Tregs can secrete soluble TGF-β and also express cell membrane–bound LAP–TGF-β (LTGF-β). Surface LTGF-β is anchored by the protein GARP. The GARP-LTGF-β complex can be activated by integrin α_v_β_8_ or α_v_β_6_ binding to the Arg-Gly-Asp (RGD) motif present in the LAP moiety of LTGF-β, which releases bioactive TGF-β. Murine and human Tregs express α_v_β_8_ that can activate GARP-LTGF-β to regulate Treg function (autocrine regulation). DCs also express α_v_β_8_ that can activate GARP-LTGF-β on Tregs to regulate Treg suppression (paracrine regulation). In addition, epithelial cells and cancer cells can express α_v_β_6_ to activate TGF-β from the GARP-LTGF-β on Tregs (paracrine regulation). Moreover, TGF-β signaling in Tregs also regulates Treg function (not depicted here), which is required for the specific suppression of Th17 cells by Tregs, yet is inhibitory for Treg suppression of Th1 cells. Abbreviations: DC, dendritic cell; GARP, glycoprotein A repetitions predominant; iTreg, induced Treg; LAP, latency-associated polypeptide; LTGF-β, latent TGF-β; SP, single positive; TAK1, TGF-β-activated kinase 1; TβR1, TGF-β receptor I; TCR, T cell receptor; TEC, thymic epithelial cell; Th1, T helper type 1; Treg, regulatory T cell; tTreg, thymic Treg. Figure adapted from images created with BioRender.com.

The function of TGF-β in regulating CD4^+^ T cell differentiation is well recognized, although knowledge of the underlying mechanisms is incomplete. It is believed that TGF-β inhibits Th1 and Th2 differentiation but promotes the generation of Th17 cells, Th9 cells, and Tregs. It is one of the most potent immunoregulatory cytokines in Th1 cell differentiation and function (Supplementary Text 3), as discussed extensively by several excellent reviews (27, 28, 30, 47, 48). TGF-β suppresses the differentiation and function of Th2 cells in vitro and in vivo (2, 48; Supplementary Text 4). The regulation of TGF-β in the differentiation and function of murine Tfh and Tfr cells is incompletely understood, but it seems to play a role in human Tfh cell differentiation, at least in culture (Supplementary Text 5). Here, I discuss the major findings and the recent advances in the field, with a focus on Th17 cells, Th9 cells, and Tregs (Figure 2).

#### 4.2.1. TGF-β in Th17 cells.

The identification and characterization of Th17 cells is a significant advance in understanding CD4^+^ T cell differentiation and function beyond the classic Th1 and Th2 paradigm (49, 50). Th17 cells produce a panel of specific cytokines, including IL-17A, IL-17F, IL-22, and IL-21, and require primarily lineage-specific transcription factor RORγt and RORα for differentiation (5, 6, 51). Although IL-23 was initially suggested to be important in the proliferation and function of Th17 cells (52), TGF-β plus IL-6 was later identified as a crucial factor for the initial differentiation of Th17 cells from naive CD4^+^ T cells (53–55). Based on the induction of Foxp3 by TGF-β in naive T cells (56), the addition of IL-6 suppresses the expression of Foxp3 and instead promotes the induction of IL-17 (53). Alternatively, coculture of Tregs with naive CD4^+^ T cells in the presence of IL-6 promotes IL-17 production, and the function of Tregs can be replaced by exogenous TGF-β (55). TGF-β is the primary factor inducing RORγt expression in CD4^+^ T cells, and this is optimized by the presence of IL-6 activating STAT3 (57, 58). Interestingly, STAT5, which is activated by IL-2, directly binds the same elements in the *Il17* gene as STAT3, displaces STAT3, and thus inhibits STAT3-mediated Th17 differentiation (59). Downstream of TGF-β signaling, activation of Smad2/3 is required for IL-17 expression (6, 51). One recent study shows that Smad4, in contrast to Smad2/3, inhibits TGF-β-induced Th17 cell differentiation by directly interacting with SKI, a transcriptional repressor that is degraded upon TGF-β stimulation (60). SKI controls histone acetylation and deacetylation of the *Rorc* locus and Th17 cell differentiation via Smad4: Ectopic SKI expression inhibits H3K9 acetylation of the *Rorc* locus, *Rorc* expression, and Th17 cell differentiation in a Smad4-dependent manner In addition, several transcriptional factors have been identified as involved in Th17 cell differentiation. For example, basic helix-loop-helix protein E2A binds to and activates the *Rorc* gene and consequently Th17 differentiation in response to TGF-β and IL-6 (61, 62). Trim33 (tripartite motif-containing 33), a modulator of TGF-β signaling that depends on Smad2, promotes the differentiation of proinflammatory Th17 cells and inhibits IL-10 (63).

Where TGF-β is produced and how it is activated for Th17 differentiation in vivo are exciting, unresolved questions. As TGF-β can be produced by almost all types of immune and nonimmune cells (16), it is likely produced in different tissues by different types of cells. For example, macrophages and immature DCs might produce and/or activate TGF-β upon phagocytosing apoptotic cells (39, 64–66). Indeed, this happens in facilitating regulatory Th17 cell differentiation in mice treated with anti-CD3 antibody, which depletes T cells during the viral infection (67). High doses of glucose can promote Th17 differentiation by activating LTGF-β through reactive oxygen species (ROS) production by T cells, thereby exacerbating the pathogenesis of Th17-mediated inflammation in mice (68). Tregs can also be a cellular source for TGF-β together with IL-6 for Th17 differentiation (55). T cell–derived TGF-β may also be a cellular source for autocrine Th17 differentiation (69).

Despite the required role of TGF-β and IL-6 in driving Th17 cell differentiation, it is evident that these Th17 cells are normally not pathogenic, which is partially due to their production of IL-10 (70). Inclusion of proinflammatory cytokines IL-23 and IL-1β enhances pathogenic function by suppressing IL-10 (71, 72). Thus, Th17 cells are proposed to comprise two functionally different subsets: nonpathogenic Th17 cells induced by TGF-β and IL-6 and pathogenic Th17 cells induced by IL-1β, IL-23, and IL-6. This notion is supported by some evidence that pathogenic Th17 cells do not to require TGF-β1 signaling (72), as CD4^+^ T cells with blockade of TGF-β signaling are still able to differentiate into pathogenic Th17 cells in response to IL-1β, IL-23, and IL-6. However, it is suggested that TGF-β3 may substitute for TGF-β1 to induce pathogenic Th17 differentiation (73). In human Th17 cells, the role of TGF-β in Th17 cell differentiation has also been debated. Some studies have shown that TGF-β is dispensable and even suppressive in human Th17 cell differentiation (74–76). However, another report argued that naive/resting CD4^+^ T cells from human blood may not be truly naive and showed that TGF-β, IL-1β and IL-6, IL-21, or IL-23 in serum-free conditions were necessary and sufficient to induce Th17 cells from naive human CD4^+^ T cells isolated from cord blood (77).

#### 4.2.2. TGF-β in Th9 cells.

Th9 cells produce IL-9 and play important roles in the pathogenesis of allergy and asthma and in antitumor immunity (7, 78). Although it was known about two decades ago that TGF-β regulated IL-9 production in CD4^+^ T cells (79), it was only in 2008 determined to be a critical factor in the differentiation of Th9 cells (80, 81). Two groups independently identified that TGF-β and IL-4 are required for *Il9* gene activation and Th9 cell differentiation. This can be accomplished either by coexposure of naive CD4^+^ T cells to TGF-β and IL-4 (80) or by TGF-β reprogramming Th2 cells to lose their characteristic profiles and switch to IL-9 secretion (81). Th9 cells do not produce IFN-γ, IL-4, or IL-17, but they may express IL-10. In addition to IL-4, IL-1α, IL-1β, IL-18, IL-33, IL-21, IL-6, and IL-10 together with TGF-β also enhance IL-9 production in CD4^+^ T cells in IL-4-dependent and -independent manners (82–84). IFN-γ and IL-27, however, are reported to inhibit Th9 differentiation (79, 84, 85). Interestingly, in polarizing conditions based on TGF-β, stimulation of OX40 promotes differentiation of naive CD4^+^ T cells toward a Th9 phenotype (86, 87), and this costimulatory effect is mediated by the TRAF6 and NF-κB pathways (87).

Human Th9 cells can also be differentiated from naive CD4^+^ T cells by TGF-β and IL-4 (82, 84, 86), although IL-6 and IL-1β are also costimulatory for Th9 cells in the presence of TGF-β (82). Human CD4^+^ memory T cells require only TGF-β to produce IL-9, as neither IL-4 nor other proinflammatory cytokines are needed (82).

Several transcription factors have been reported to participate in the downstream signaling of TGF-β and IL-4 to activate *Il9* gene expression, including PU.1, IRF4, E2A, Stat5, Stat6, GATA3, BATF, Smad2/3, and NF-κB (88). However, none of these transcription factors has been recognized as the lineage-specific transcription factor for Th9 cells, because they are also involved in the differentiation of other Th cells. It is reported that PU.1, IRF4, BATF, and GATA3 are all regulated by TGF-β signaling (88). It is possible that multiple transcription factors, rather than a specific single one, are required for Th9 differentiation, or that the specific transcription factor has not yet been identified. Immediately downstream of TGF-β signaling, Smad2, Smad3, and Smad4 are reported to be involved in Th9 cell differentiation (86, 89–91). It is suggested that Smad2/4 may promote *Il9* gene expression through EZH2 displacement (91), and that Smad2/3 and IRF4 cooperate in Th9 induction (90), but how these Smads function as direct or indirect mediators in *Il9* gene transcription is still largely unknown. In addition, TGF-β may also signal to activate the *Il9* gene through a Smad3-independent pathway. For example, it is reported that TGF-β and IL-4 activate TAK1, a critical component of the non-Smad-dependent pathways (35, 36), which downregulates Id3 expression and consequently promotes E2A and GATA3 binding to the *Il9* gene to activate its transcription (86).

It should be pointed out that although it is well established that Th9 cells can be differentiated in cell culture, Th9 cells are hardly detectable in the tissues in situ (92). This raises the question as to whether Th9 cells are only transiently present in vivo and transduce signaling to other immune cells, or whether they switch into a different population of T cells. This question is also related to the functional mechanisms by which Th9 cells carry out their antitumor activities and immunopathogenic effects with respect to allergy and asthma.

#### 4.2.3. TGF-β in Tregs.

CD4^+^CD25^+^Foxp3^+^ Tregs are essential to induction and maintenance of immune homeostasis and tolerance (8). Tregs regulate the immune responses in autoimmunity, inflammation, transplant rejection, allergy/asthma, infections, and cancer. They can develop in the thymus as natural, or thymic, Tregs (tTregs) and can also be generated in the periphery by conversion from CD4^+^Foxp3^−^ naive T cells, in which case they are called induced Tregs (iTregs) or peripheral Tregs (pTregs). Foxp3 is the lineage-specific transcription factor for Tregs and controls their development (93–95). TGF-β plays a key role in the induction of Foxp3 and the development of and function of both iTregs and tTregs (32, 37, 56, 96), and this role is the focus of discussion in this section (Figure 2).

##### 4.2.3.1. TGF-β in induced/peripheral Tregs.

The identification and characterization of CD4^+^CD25^+^ Tregs by Sakaguchi et al. (97) has revolutionized our understanding of immunoregulation. Because Tregs freshly isolated from spleens and lymph nodes in normal mice not only are anergic to TCR stimulation but also exhibit potent suppressive activity against CD4^+^CD25^−^ responder T cells in an in vitro suppression assay (98), Tregs were once called CD4^+^CD25^+^CTLA-4^+^ anergic/suppressor T cells. It was once thought that Tregs only developed in the thymus, and not converted from naive CD4^+^ T cells in the periphery. Based on our longstanding interest in TGF-β regulation of T cells, in 2001 we discovered that TGF-β induces CD4^+^CD25^+^CTLA-4^+^ anergic/suppressor T cells from murine peripheral naive CD4^+^CD25^−^ T cells in the context of TCR stimulation in vitro (99). However, another group reported that in vitro stimulation of human blood CD4^+^ T cells with irradiated allogeneic peripheral blood mononuclear cells in the presence of TGF-β led to expansion of existing CD25^+^ cells rather than converted CD25^+^ cells from CD4^+^CD25^−^ T cells (100). Immediately after the discovery that Foxp3 is the key and lineage-specific transcription factor for the development and function of CD4^+^CD25^+^ Tregs (93–95), we discovered that TGF-β, in the context of TCR stimulation, induces *foxp3* gene expression from peripheral naive CD4^+^CD25^−^ T cells and converts them into CD4^+^CD25^+^Foxp3^+^ Tregs (56), a finding subsequently reported by numerous independent groups (101–105). Tregs were soon reported to have been converted from naive CD4^+^CD25^−^ T cells in vivo by using a TCR transgenic CD4^+^ T cell adoptive transfer system, or in non-transgenic settings in a TGF-β-dependent manner (65, 96, 106–109). For example, weak TCR stimulation with minute antigen doses and suboptimal DC activation favors Treg generation in a TGF-β-dependent manner (96), but this only occurs in naive mice. In gut-associated lymphoid tissues (GALTs), which are enriched with TGF-β, retinoic acid that can be produced by CD103^+^ DCs enhances TGF-β-induced Treg conversion (107, 109, 110). The successful conversion of Tregs from naive CD4^+^ T cells in vitro and in vivo by TGF-β has not only proven that *foxp3* can be induced from naive CD4^+^ T cells; it has also opened up a way to induce antigen-specific Tregs for potential immunotherapy for autoimmune diseases, allergy/asthma, and transplantation.

Indeed, efforts have been made to develop immunotherapy to experimental autoimmunity and allergy by manipulating TGF-β and Tregs. Adoptive transfer of in vitro induced antigen-specific Tregs by TGF-β could potentially be used to treat autoimmunity. However, it was reported that iTregs are less stable based on their DNA-demethylation status (111) and may be susceptible to losing their Foxp3 expression in vivo. Thus, several approaches have been developed to enhance the stability of iTregs. For example, vitamin C potentiates ten-eleven translocation (TET) activity and acts through Tet2/Tet3 to increase the stability of Foxp3 expression in TGF-β-induced Tregs (112). Whole-genome analyses show that the addition of vitamin C during TGF-β-induced iTreg differentiation in vitro potentiates the expression of Treg signature genes and alters the epigenetic landscape to better resemble that of Tregs generated in vivo (113).

On the other hand, progress has been made in inducing antigen-specific Tregs in vivo by manipulating TGF-β, especially in mice with established autoimmunity. Despite the success of iTreg conversion in naive mice (96), inducing antigen-specific Tregs in mice with established autoimmunity was unsuccessful until only recently. Based on the findings that phagocytes produce and secrete TGF-β upon engulfment and digestion of apoptotic cells in vitro and in vivo (64, 66, 114), and that TGF-β is essential for the conversion of naive T cells into Tregs in the presence of TCR stimulation (56), we have successfully developed an experimental approach to induce autoantigen-specific Tregs in mice with autoimmune diseases. This protocol includes three functional steps, namely induction of a sufficient number of apoptotic immune cells such as T and B cells; contact and digestion of apoptotic cells by phagocytes to produce TGF-β; and subsequent administration of low doses of autoantigenic peptides (TCR stimulation) in mice with disease. These in vivo induced autoantigen-specific Tregs potently suppress autoimmunity and inflammation in a variety of experimental autoimmune disease models, including models of experimental autoimmune encephalitis, experimental autoimmune uveitis, type 1 diabetes, and Sjögren syndrome (65, 115–117). Importantly, this treatment does not compromise immunity to bacterial antigens and tumors in mice (65, 116). In line with this, oral administration of hyperphysiological doses of d-mannose, an epimer of glucose, can suppress type 1 diabetes and asthmatic lung inflammation by inducing Tregs from naive CD4^+^ T cells in mice (118). Mechanistically, D-mannose can activate LTGF-β by upregulating integrin α_v_β_8_ and increasing ROS production in T cells.

Generation of human CD4^+^CD25^+^Foxp3^+^ iTregs also requires TGF-β and IL-2 in addition to TCR stimulation (102, 104), although their in vitro immunosuppressive activity may vary (119). It was suggested that human Foxp3^+^ T cells can be induced in CD4^+^Foxp3^−^ T cells by TCR stimulation in the absence of exogenous TGF-β, but more careful analyses revealed that these TCR-driven Foxp3^+^ T cells from CD4^+^CD25^−^Foxp3^−^ T cells still require production and/or activation of TGF-β in serum-containing culture medium or in T cells (120, 121).

##### 4.2.3.2. TGF-β in thymic Tregs.

TGF-β signaling is also required for the development of tTregs in the thymus (37). Early studies suggested that TGF-β signaling might be dispensable for the development of tTregs, as adult transgenic mice with specific deletion of TGFβRII in T cells (TβRII^ko^) did not show a significant reduction (22, 24), and actually showed an increase, in the frequency of tTregs (22). However, subsequent studies in mice with T cell–specific deletion of TGFβRI (TβRI^ko^) revealed that a deficiency of TGF-β signaling in the thymocytes results in a profound defect of tTregs in the thymus during the neonatal stage (days 3–5) without significant changes in other populations of thymocytes (23). This was confirmed by another independent study with TβRII^ko^ mice (40). Consistent with an early report (22), TβRI^ko^ mice also gradually accumulated tTregs in the thymus with age, and by 2–3 weeks the frequency of tTregs in the knockout mice was actually higher than that in the age-matched wild-type mice (23). This paradox was resolved by the findings that deletion of IL-2 in TβRI^ko^ mice (IL-2^−/−^TβRI^ko^) completely abolished the increase in tTregs observed in the single-knockout TβRI^ko^ mice and that the double-knockout mice showed a profound decrease in tTregs, from neonatal through adult ages (23). Thus, the increased frequency of tTregs in the adult TβRI^ko^ mice is due to the accelerated expansion of a few tTregs driven by increased IL-2 in the TβRI^ko^ thymus. Thus, the crucial role of TGF-β signaling in the development of tTregs is now recognized (37, 38).

An important question, then, is the underlying mechanisms by which TGF-β controls tTreg development. Based on ample evidence that TGF-β is essential for *foxp3* gene transcription in naive CD4^+^ T cells and also in CD4^+^ SP thymocytes (39, 56), it is conceivable that the defect in tTregs in TGFβR knockout mice is due to the lack of TGF-β induction of the *foxp3* gene in thymic CD4^+^ SP precursors. However, a study suggested that the defect of tTregs in the TβRII^ko^ thymus was due to increased death of tTregs specifically in the absence of TGF-β signaling in T cells (40). While it is well known that TGF-β signaling is indeed important to protect thymocytes from unwanted death (122), this protection is unlikely unique to tTregs. Indeed, when TβRI was deleted only after Foxp3^+^ was expressed in tTregs (*Tgfbr1*^flox/flox^*Foxp3-Cre*^+^), there was no difference in either the frequency or the absolute number of tTregs at neonatal age or in adult mice (39), eliminating the specific TGF-β protection of tTregs as the major mechanism for the deficiency of tTregs in the knockout mice. Through multiple experimental approaches involving in vitro FOTC (fetal organ thymus culture) cultures and in vivo intrathymic injections, in both normal and TCR transgenic mice, Konkel et al. (39) provided indisputable evidence that TGF-β signaling is indeed required for *foxp3* gene induction in CD4^+^ SP thymocytes. In understanding where and how TGF-β is produced and/or activated, we discovered that the apoptosis of thymocytes could drive thymic macrophages, DCs, and thymic epithelial cells to produce TGF-β that can be activated by as yet identified mechanisms (39). These findings prompt us to propose a modified model to explain the development of tTregs by linking thymic apoptosis (e.g., negative selection) to the development of tTregs in a TGF-β-dependent manner (Figure 2). This model could reconcile well with the current models of tTreg development (Supplementary Text 6).

The molecular mechanisms underlying TGF-β induction of *foxp3* in the tTreg SP precursors are still incompletely understood. The *foxp3* gene contains the conserved promoter sequence, located upstream of the transcriptional start site, and conserved noncoding sequence 0 (CNS0), CNS1, CNS2, and CNS3 (123–129). Downstream of TGF-β receptors, pSmad2 and pSmad3 are crucial for *foxp3* gene activation (25, 128). Smad3 may directly bind to CNS1 together with TCR signaling–induced NFAT to activate *foxp3* transcription (128), which was suggested to be responsible for iTreg/pTreg generation (129). An earlier argument against a role of TGF-β in tTreg development was based on the findings that there are no obvious Smad3-binding sequences in the *foxp3* promoter or in CNS3, which were proposed to be involved in the induction of tTregs (128, 129). However, recent studies have revealed that CNS0 is even more important than CNS3 in initiating *foxp3* transcription in tTregs (123, 126, 130). Binding of pStat5 to CNS0 is suggested to be the key factor in initiating *foxp3* transcription, and it is believed that pStat5 is activated by IL-2. However, it remains to be known whether the expression and activation of Stat5 in CD4^+^Foxp3^−^ SP thymocytes require TGF-β signaling.

Furthermore, even if there is no obvious Smad-binding site(s) in the *foxp3* promoter or in CNS0 or CNS3, it is still possible that Smad can, through interaction with copartners, indirectly bind to and regulate *foxp3* expression. Indeed, it has been shown that Smad3 binds at the *foxp3* promoter and regulates its activity, through an “enhancersome,” with other transcription factors like cRel that can directly bind the promoter and CNS3 (127). Thus, it might be time to reconsider the possibility that Tregs are all generated via similar signaling pathways. The major difference between tTregs and iTregs/pTregs would therefore be the location in which the Treg is generated and, as such, the antigens driving their development, rather than the requirement for TGF-β signaling (37).

##### 4.2.3.3. TGF-β in Treg function.

The function of TGF-β in Treg-mediated immunosuppression is still incompletely understood. TGF-β-mediated Treg suppression can be classified into three types, namely autocrine TGF-β production and activation by Tregs, paracrine TGF-β production and/or activation between Tregs and other cells, and the effects of TGF-β signaling in Tregs on their suppression. Tregs can secrete a soluble form and express a cell membrane–bound form of TGF-β (32, 33, 131–133). However, the exact function of autocrine TGF-β in Treg-mediated suppression still remains controversial.

To better understand the role of autocrine TGF-β production by Tregs in mediating their immunoregulation, several groups have generated T cell–specific TGF-β1 knockout mice (69, 134–137). However, these transgenic mice have generated more conflicting results. In a recent excellent review, Moreau et al. (32) carefully analyzed the contradictory phenotypes of the mice and explained that they are due to technical differences and some potential off-target effects during the generation of the T cell–specific *Tgfb1*^−/−^ mice. They believe that the evidence favors the notion that Treg-mediated immunosuppression is largely impervious to the loss of endogenous TGF-β under baseline conditions, and they conclude that Treg-derived TGF-β is dispensable for Treg-mediated immune suppression and that self-tolerance can be maintained in the absence of Treg-produced TGF-β (32). However, the potential roles of Treg-derived TGF-β in conferring Treg-mediated suppression in pathogenic conditions such as autoimmunity, chronic inflammation, cancer, and infection still cannot be excluded.

The identification of GARP (glycoprotein A repetitions predominant) as an anchor that complexes with LTGF-β on the surface of murine and human Tregs provided a structural basis for the early findings of cell membrane–bound LAP-TGF-β on Tregs (131, 132), which is required for the activation of LTGF-β to mediate Treg function in an autocrine or paracrine manner (32, 33, 133). This can be accomplished by integrin α_v_β_8_ or α_v_β_6_ binding to the tripeptide Arg-Gly-Asp (RGD) motif present in the LAP moiety of LTGF-β (138). Both murine and human Tregs express α_v_β_8_ that activates TGF-β to increase in pSmad2 in Tregs or to induce Foxp3 from cocultured naive CD4^+^ T cells in vitro (133, 139). The GARP-LTGF-β complex can also be activated by interactions with the α_v_β_8_ in DCs to induce iTregs from naive CD4^+^ T cells or to suppress other immune cells (140). In addition, GARP can also be proteolytically cleaved by thrombin and platelets to release active TGF-β1 for cancer immune evasion (141). Moreover, the GARP-LTGF-β complex on Tregs may also be activated by α_v_β_6_ on epithelial cells to regulate immune and nonimmune cells, which may be involved in tissue repair and wound healing or even fibrosis (32, 33) (Figure 2).

Finally, TGF-β signaling in Tregs also plays a role in Treg-mediated immunoregulation. It is generally believed that Tregs regulate immune responses through at least two functional mechanisms: general suppression of the activation and proliferation of T cells and other immune cells and specific regulation of individual Th subsets. For example, Treg expression of T-bet is required for suppression of Th1 responses (142), and Stat3 for suppression of Th17 responses (143). By generating transgenic mice that lack TβRI specifically in Foxp3^+^ Tregs, we revealed that deletion of TGF-β signaling in Tregs does not compromise their general suppressive activity with respect to T cell proliferation (144). However, these TβRI knockout Tregs show increased suppressive function against Th1 cells by expressing higher levels of T-bet and CRCX3, but they exhibit defective suppressive activity toward Th17 cells by an as yet unidentified mechanism (144). These findings indicate that TGF-β signaling in Tregs is dispensable for the general suppressive activity against T cell proliferation but is required for the specific suppression of Th17 cells, yet inhibitory for Treg suppression of Th1 cells. Unexpectedly, these TβRI^ko^ Treg mice exhibit a specific defect in the recruitment and retention of Tregs in the gastrointestinal tract past 6 months, resulting in an inability to regulate inflammation in the gut. This is ascribed to the lack of CD103 expression in the TβRI^ko^ Tregs (144).

## 5. TGF-β IN CD8^+^ T CELLS

Based on their phenotypic, functional, transcriptional, and epigenetic state, CD8^+^ T cells can be classified into naive, effector (Teff ), exhausted (Tex), and memory cells. Memory T cells can be further classified into circulating effector memory T (Tem) and central memory T (Tcm) and non-circulating and tissue-resident memory (Trm) cells (10, 11, 145). TGF-β regulates the activation, proliferation, differentiation, and function of CD8^+^ T cells. Also, TGF-β appears to play a role in their generation of a unique population, CD8^+^Foxp3^−^CD122^+^Ly49^+^ Tregs (146, 147). In this section, I highlight the recent findings and advances in TGF-β regulation of CD8^+^ T cells with emphasis on Teff, Trm, Tex, and CD8^+^ Tregs (Supplementary Figure 1).

### 5.1. TGF-β Suppression of Effector CD8^+^ T Cells

TGF-β suppresses CD8^+^ T cell activation and function through direct and indirect pathways. CD8^+^ T cells are major cytotoxic T lymphocytes that produce IFN-γ and express granzyme A and B and perforin to kill their target cells, especially tumor cells and virus-infected cells (48). In vitro, TGF-β suppresses the activation and differentiation of murine and human naive CD8^+^ T cells isolated from cord blood to effector CD8^+^ T cells (148, 149). The regulatory role of TGF-β in the CD8^+^ effector function is best evidenced by a variety of in vivo models of tumor, infection, and inflammation in mice in which TGF-β signaling is altered by either genetic manipulation or inhibition with antibodies or inhibitors. dnTβRII (dominant-negative TGF-β receptor II) mice exhibit a large expansion of tumor-reactive CD8^+^ T cells that produce high amounts of IFN-γ and granzyme B and eradicate tumors (48). The suppressive activity of TGF-β in antitumor CD8^+^ Teff cells can be performed either by specifically inhibiting the expression of perforin, granzymes A and B, Fas ligand, and IFN-γ in a Smad-dependent manner (150) or by indirect suppression through Treg-derived cell membrane–bound TGF-β (151) and via exclusion of CD8^+^ T cell infiltration to the tumor (152, 153). In addition, TGF-β enhances antigen-induced PD-1 expression through Smad3-dependent transcriptional activation in T cells in vitro and in tumor-infiltrating lymphocytes in vivo, which impedes antitumor activity (154). Systemic blockade of TGF-β with anti-TGF-β antibodies in combination with DNA vaccination or IL-2 treatment enhances tumor-infiltrating and tumor-reactive CD8^+^ T cells (155, 156). Moreover, TGF-β might also control effector cell number by lowering BCL-2 amounts and promoting apoptosis of short-lived effector cells in mice, which interferes with anti-infection immunity in *Listeria* infection (157) and promotes medulloblastoma progression in SmoA1 (smoothened A1) transgenic medulloblastoma mice (158). In nonobese diabetic (NOD) mice, transgenic expression of TGF-β in the inflamed islets significantly delays diabetes development, in that TGF-β disables the transition of primed autoreactive CD8^+^ T cells to cytotoxic effectors within the pancreas, significantly impairing their diabetogenic capacity (159). Transgenic mice with T cell–specific deletion of TGF-β receptors or Smad2/3 results in massive CD8^+^ Teff cell activation and large amounts of IFN-γ secretion (22–25). In contrast to the CD4-Cre-driven system, adult mice with deleted TβRII, through the use of Cre driven by a promoter (dLck-Cre) that is active much later in T cell development, exhibit no obvious sign of autoimmunity or systemic inflammation and have milder activation of CD8^+^ T cells (160). However, adoptive transfer of these knockout T cells into lymphopenic hosts results in inflammation. Zhang & Bevan (160) propose that TGF-β may mainly regulate T cell proliferation and activation in response to exogenous stimuli, such as lymphopenia. However, an alternative explanation is that the late deletion of TβRII in T cells may spare TGF-β control of the development of tTregs in the neonatal window and that Tregs in turn function normally in maintaining immune homeostasis and tolerance in these knockout mice.

### 5.2. TGF-β in CD8^+^ Resident Memory T Cells

CD8^+^ Trm cells reside permanently in nonlymphoid tissues and appear phenotypically, functionally, transcriptionally, and metabolically different from Tcm and Tem cells. Trm cells lack CCR7 and CD62L and thus are unable to recirculate through the blood and lymphoid organs; instead, they express unique clusters of molecules that safeguard their residence in nonlymphoid tissues. This includes CD69, which inhibits sphingosine-1 phosphate receptor (SIPR1) to prevent Trm cells from egressing; CD103 (α_E_β_7_), an integrin that binds to E-cadherin in epithelial cells; and CD49a, which tethers Trm cells to be retained in the tissues of residence (161, 162). Functionally, Trm cells are major CD8^+^ T cells in the tissues for the defense against infections and malignant cells; they produce IFN-γ and TNF-α and express cytotoxic molecules such as granzyme B to kill their target cells, although their expression may vary in different tissues (10).

TGF-β plays a central role in the differentiation and maintenance of Trm cells. Trm cells can be differentiated from circulating naive and memory precursors and/or from effector or memory cells in nonlymphoid tissues. Upon antigen activation, CD8^+^ T cells can be differentiated into either short-lived Teff cells (CD127^−^KLRG-1^+^) or memory precursor Teff cells (CD127^+^KLRG-1^−^) (157, 163). CD8^+^ T cells downregulate TGF-β receptors upon TCR stimulation (46, 164), but extracellular ATP senses the purinergic receptor P2RX7 on CD8^+^ T cells to help them regain TGF-β receptors and restore their sensitivity to TGF-β, which promotes differentiation of memory precursor Teff cells to CD103^+^CD8^+^ Trm cells (164).

TGF-β induces CD103 in CD8^+^ Trm cells through Smad3 binding to the *Itgae* gene. In addition, TGF-β also indirectly upregulates CD103 expression by suppressing T-bet and TCF-1 expression, as both may bind to *Itgae* and interfere with Smad3-mediated CD103 gene transcription (165, 166). However, CD103^+^ Trm cells appear to be tissue specific, as liver CD8^+^ Trm cells do not express CD103 (145). Moreover, CD103^−^CD8^+^ Trm cells in the gut lamina propria do not require TGF-β, as TGF-β receptor knockout mice show no effects on these CD103^−^ Trm cells (167). Interestingly, TGF-β is also required for the formation of CD69^+^CD103^+^ Trm cells in the corneal epithelium, and these Trm cells patrol the cornea (168). In addition to CD103, TGF-β also enhances expression of CD49a in CD8^+^ T cells during antigen stimulation, such as in infections; however, CD49a is lost in most circulating memory T cells but is maintained in many Trm cells (169).

TGF-β is also critical in the suppression of Trm cell egress from the tissues. S1PR1 is a central molecule that promotes the egress of CD8^+^ Trm cells from the tissues (170). TGF-β suppresses SIPR1 expression in Trm cells through different mechanisms. TGF-β alone or in combination with IL-33 suppresses KLF2 through the PI3K/AKT pathway, which consequently inhibits S1PR1 expression, as KLF2 binds to the *Sipr1* gene promoter to promote *Sipr1* transcription. In addition, TGF-β also downregulates SIPR1 by upregulating CD69 in CD8 Trm cells (167).

The accumulated evidence indicates that integrin-mediated activation of LTGF-β is probably a major source of bioactive TGF-β that regulates Trm cells in the tissues. For example, in lymph nodes, not in the spleen, naive CD8^+^ T cells are conditioned through MHC-I-dependent interactions with peripheral tissue-derived migratory DCs that express α_v_β_8_ to activate TGF-β1, and this induces epithelial CD8^+^ Trm cells (171). In the tumor microenvironment, it is reported that type 1 Tregs express high amounts of α_v_β_8_ integrin to activate TGF-β and promote Trm cell differentiation (172). Even tumor cells can also express α_v_β_6_ integrin to activate TGF-β and enhance CD103^+^CD69^+^CD8^+^ Trm cells, and these Trm cells may interfere with anti-PD-1/anti-PD-L1-mediated immunotherapeutic effects (173).

### 5.3. TGF-β in CD8^+^ Exhausted T Cells

During chronic viral infections and cancer, CD8^+^ T cells can become Tex cells. CD8^+^ Tex cells exhibit defective production of IFN-γ and TNF-α cytokines; are marked with some group of surface molecules, such as PD-1, Tim3, LAG3, CD73, and 2B4 (174); and become dysfunctional and fail to form immunity against infections and cancer. Recent studies have provided evidence that TGF-β might also be involved in CD8^+^ T cell exhaustion during chronic infection and cancer. A breast cancer study showed that breast cancer cells secrete extracellular vesicles in the form of exosomes that carry PD-L1 and are highly immunosuppressive. TGF-β promotes breast cancer exosomal PD-L1 secretion, and this process facilitates CD8^+^ T cell dysfunction and exhaustion (175). Comparison of the transcriptome of “exhausted” CD8^+^ T cells infiltrating autochthonous melanomas to those of naive and acutely stimulated CD8^+^ T cells reveals that the transcriptional regulators Nr4A2 (nuclear receptor subfamily 4 group A member 2) and Maf are overexpressed in tumor Tex cells and are significantly upregulated in CD8^+^ T cells from human melanoma metastases. TGF-β and IL-6 are the main inducers of Maf expression in CD8^+^ T cells (176). In a model of chronic persistent murine cytomegalovirus infection, CD8^+^ T cells appear exhausted, expressing PD-1, CD73, and CD39, and intriguingly, CD73 is activated on CD8^+^ T cells by TGF-β signaling (177). However, still other studies show that TGF-β-mediated dysregulation of CD8^+^ T cells may not necessarily be due to induction of exhaustion. For example, in a tumor model, TGF-β induces Foxp1 expression through Smad2/3-mediated c-myc repression, which prevents CD8^+^ T cell activation by tumor antigens, and this process is not associated with T cell exhaustion (178). Similarly, in a chronic virus infection model, it was shown that CD8^+^ T cell–intrinsic TGF-β signaling was responsible for virus-specific CD8^+^ T cell apoptosis and decreased cell numbers but was not necessary for their functional exhaustion (179). Nevertheless, a recent study showed that TGF-β repressed mTOR signaling in CD8^+^ Tex cells and was a critical determinant of the metabolism and function of precursors of Tex cells. These Tex cell precursors sustained Tex cells and self-renewed while continuously generating exhausted effector T cells (180). This underlines the metabolic involvement of TGF-β regulation of CD8^+^ T exhaustion.

### 5.4. TGF-β in CD8^+^ Tregs

Naive CD8^+^ T cells can also differentiate into Foxp3^+^ T cells in response to TCR stimulation in the presence of TGF-β (181). These Tregs manifest equal, if not stronger, suppressive activity with respect to CD4^+^Foxp3^+^ Tregs to inhibit immune cell activation and proliferation in coculture assays in vitro. However, it is mysterious that CD8^+^Foxp3^+^ Tregs are hardly detectable in normal mice in the steady state. This raises the question as to whether CD8^+^Foxp3^+^ Tregs even exist in mice and humans. About ten years ago, Kim et al. (182) reported on a population of CD8^+^ Tregs that expressed a unique set of molecules, namely CD122 and Ly49, but remained Foxp3^−^. Importantly, a more recent study indicated that the human counterparts of murine CD8^+^ Tregs have been discovered and are more numerous in those with autoimmune diseases such as systemic lupus erythematosus, Crohn disease, and multiple sclerosis as well as in patients infected with influenza virus or SARS-CoV-2 (183). These human CD8^+^ Tregs express a unique killer immunoglobulin receptor as well as Helios, which is also found in murine CD8^+^ Tregs. Of note, recent studies suggest that TGF-β is critical in the differentiation of CD8^+^ Tregs in mice (146, 147). In one study investigators generated TβRII and Eomes double-knockout mice (*Tgfbr2*^flox/flox^/*Eomes*^flox/flox^*dlck-Cre*) and demonstrated that these mice specifically lack CD8^+^Foxp3^–^CD122^+^Ly49^+^ Tregs, whereas CD4^+^Foxp3^+^ Tregs and Tfr cells are not affected. Mechanistically, TGF-β induces and upregulates Helios in CD8^+^ T cells but not CD4^+^ T cells, although this has not been explained. In addition, TGF-β plus Eomes can preserve CD122 expression in these CD8^+^ Tregs (146).

## 6. TGF-β IN *γ*δ T CELLS

Recent studies have reported that TGF-β plays a role in the differentiation of IL-17-producing γδ T cells and IL-9-producing Vδ2 γδT cells. In normal mice, IL-17^+^ γδT cells are an important cellular source of IL-17 and are involved in defense against bacterial infection and in autoimmune inflammation. TGF-β controls in the development of IL-17^+^ γδT cells in the thymus (184). Interestingly, γδ T cells, by secreting IL-17F, drive adipocytes to express TGF-β via IL-17 receptor C (IL-17RC), and TGF-β promotes local sympathetic innervation to enhance thermogenesis (185). Human Vδ2 T cells are the dominant γδ T cell subset found in peripheral blood and recognize pyrophosphate molecules derived from microbes or tumor cells. TGF-β, in the presence of IL-15, induces Foxp3^+^ Vδ2 T cells that show some suppressive activity toward CD4^+^ T cells. However, these Vδ2 T cells express high amounts of the IL-9 gene (186).

## 7. TGF-β IN NATURAL KILLER T CELLS

**Figure 3:**
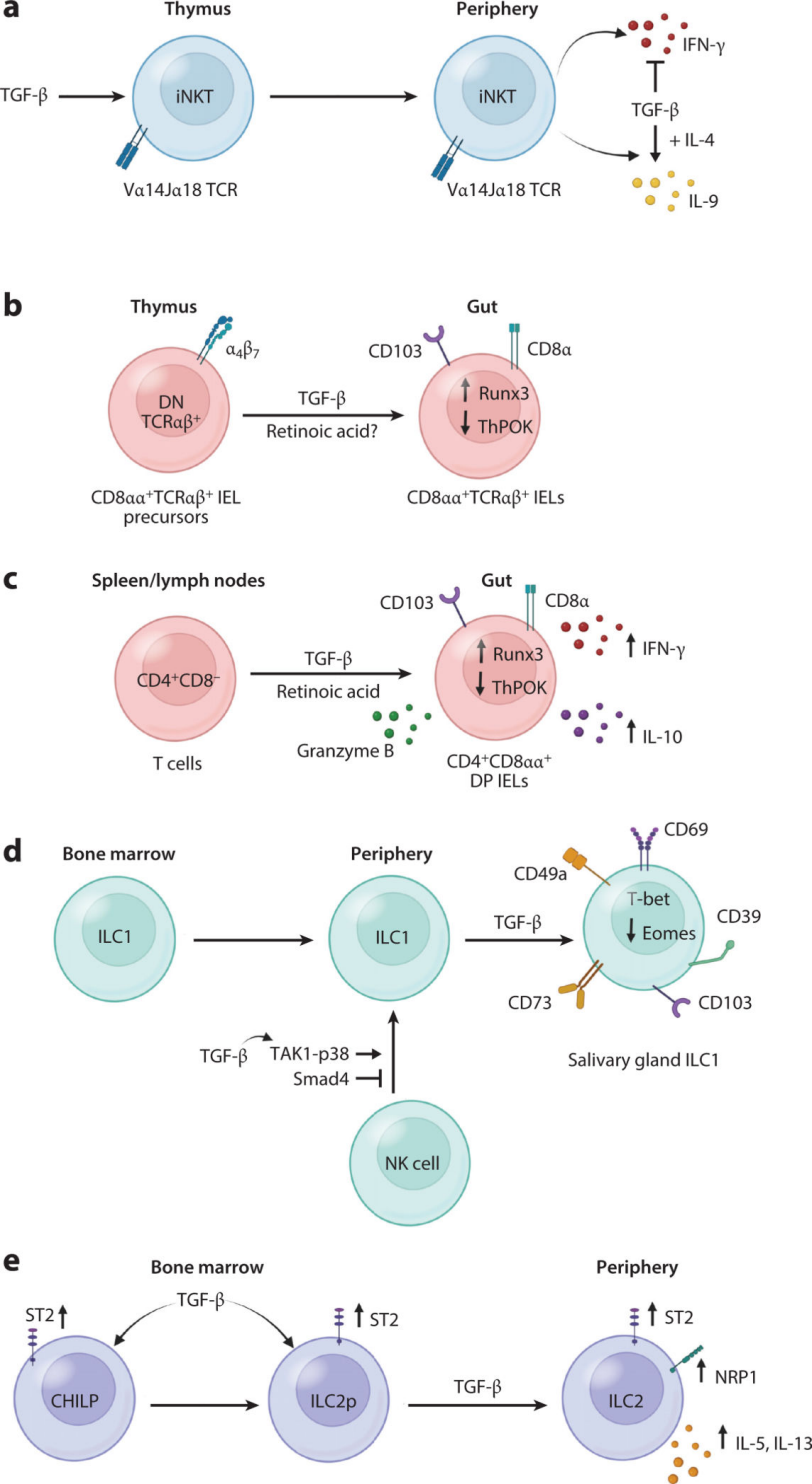
TGF-β regulation of unconventional lymphocytes. (*a*) In the thymus, TGF-β regulates the development of iNKT cells. In the periphery, TGF-β represses their ability to produce IFN-γ, while, in combination with IL-4, promoting their ability to produce IL-9. (*b*) TGF-β is a key factor to control the development and maintenance of TCRαβ^+^CD8αα^+^ IELs. It protects DN (CD4^−^CD8^−^) TCRαβ^+^CD5^+^ thymocytes (the precursors of TCRαβ^+^CD8αα^+^ IELs) from apoptosis. TGF-β also induces the expression of CD8α via downregulation of ThPOK and upregulation of Runx3 in the precursors. (*c*) TGF-β induces, together with retinoic acid, the generation of CD4^+^CD8αα^+^ DP IELs by inducing CD8α expression in peripheral CD4^+^CD8^−^ T cells, and this is accomplished by suppressing ThPOK and upregulating RUNX3 expression. TGF-β also induces the expression of CD103. CD4^+^CD8αα^+^ DP IELs produce IL-10 and IFN-γ, and thus are considered cytotoxic T cells with regulatory function. (*d*) TGF-β promotes the differentiation of ILC1s in the salivary glands by suppressing Eomes, through Junk-dependent but Smad4-independent pathways. TGF-β also promotes the conversion of NK cells into ILC1s through the TAK1-p38 pathway, and Smad4 is inhibitory for this conversion. (*e*) In the bone marrow, TGF-β programs the development of ILC2s by upregulating the expression of ST2 in the CHILPs and the ILC2 precursors. TGF-β also maintains ST2 expression in mature ILC2s. Moreover, TGF-β induces Nrp1 expression in lung ILC2s further enhancing ILC2s functions by increasing ST2 expression. Abbreviations: CHILP, common helper-like innate lymphoid progenitors; DN, double negative; DP, double positive; IEL, intraepithelial lymphocyte; ILC1, group 1 innate lymphoid cell; ILC2p, ILC2 precursor; iNKT, invariant NK T; NK, natural killer; TAK1, TGF-β-activated kinase 1; TCR, T cell receptor. Figure adapted from images created with BioRender.com.

NKT cells recognize self–glycolipid antigens and foreign glycolipid antigens presented by the nonclassical MHC-I-like molecule CD1d (187). NKT cells comprise two subsets, namely iNKT cells and type II NKT cells. iNKT cells utilize a semi-invariant TCR involving Vα14Jα18 in mice and Vα24Jα18 in humans (30, 47) to respond to α-galactosylceramide bound to CD1d. iNKT cells develop in the thymus, and ablation of TGF-β receptors in T cells block development of canonical CD1d-restricted NKT cells (24). However, the underlying mechanisms by which TGF-β controls the development of iNKT cells remains incompletely understood. CD1d^−/−^dnTβRII mice, which lack CD1d-restricted NKT cells, exhibit significantly decreased hepatic lymphoid cell infiltrates and milder cholangitis compared with CD1d^+/−^dnTβRII mice (188). In contrast to its positive role in the development of iNKT cells, TGF-β actually suppresses iNKT cell functions, such as IFN-γ production (30, 47). Interestingly, IL-9 production by iNKT cells is not imprinted during thymic development but is rather induced by TGF-β and IL-4 stimulation from mature peripheral iNKT cells (189) (Figure 3).

## 8. TGF-β IN INTRAEPITHELIAL LYMPHOCYTES

Intraepithelial lymphocytes (IELs) comprise heterogeneous populations of lymphocytes residing between intraepithelial cells (13, 190). Unconventional IELs (also called natural IELs) comprise TCRαβ^+^CD8αα^+^ and TCRγδ^+^CD8αα^+^ populations that develop in the thymus from CD4^+low^CD8^+low^Pd1^hi^ through TCRαβ^+^CD8^−^CD4^−^ α_4_β_7_^+^ thymocytes and from CD4^−^CD8^−^TCRVγ7^+^ thymocytes, respectively (190). Conventional IELs (also called induced IELs) include CD4^+^CD8^−^ and CD4^−^CD8αβ^+^TCRαβ^+^ IELs and CD4^+^CD8αα^+^TCRαβ^+^ cells (DP IELs). TGF-β plays a critical role in the development, differentiation, and maintenance of TCRαβ^+^CD8αα^+^ and TCRαβ^+^CD4^+^CD8αα^+^ DP IELs (Figure 3).

### 8.1. TGF-β in TCRαβ^+^CD8αα^+^ IELs

TGF-β is a key factor to control the development and maintenance of TCRαβ^+^CD8αα^+^ IELs, because transgenic mice with either *Tgfb1*^−/−^ or T cell–specific TGFβRI deficiency (TβRI^ko^) lack TCRαβ^+^CD8αα^+^ IELs (41). In addition, lack of Smad3 also results in a significant reduction of TCRαβ^+^CD8αα^+^ IELs in mice. Conversely, mice with overexpression of active TGF-β1 specifically in T cells (*Tgfb1glo*^+^*CD4cre*^+^) have more TCRαβ^+^CD8αα^+^ IELs (41). Intriguingly, conventional TCRαβ^+^CD8αβ^+^ IELs are not affected, and actually their frequency is increased in mice with TGF-β signaling deficiency. TGF-β protects DN TCRαβ^+^CD5^+^ thymocytes, the immediate precursors of TCRαβ^+^CD8αα^+^ IELs (13), from apoptosis (41). Once leaving the thymus, these IEL precursors express α_4_β_7_ integrin and CCR9, leading them to the gut epithelium (190), where on exposure to TGF-β they acquire expression of CD8α and CD103 (α_E_β_7_) (41, 191), but downregulate the expression of α_4_β_7_ (192). TGF-β induction of CD8α in TCRαβ^+^CD8αα^+^ IEL precursors is mediated by downregulation of ThPOK leading to a reduced ratio of ThPOK to RUNX3 (41). In addition, TGF-β also maintains CD8α expression on TCRαβ^+^CD8αα^+^ IELs. A recent report provided some evidence that humans also have CD8αα^+^TCRαβ^+^ intestinal IELs (193), although the role of TGF-β in their development remains to be shown.

### 8.2. TGF-β in CD4^+^CD8α^+^ Double-Positive IELs

TCRαβ^+^CD4^+^CD8αα^+^ DP IELs are also called induced IELs, as they are derived from peripheral CD4^+^CD8^−^ T cells in the lymphoid tissues (194, 195). The first evidence supporting the notion that DP IELs are converted from CD4^+^CD8^−^ T cells is that adoptive transfer of peripheral CD4^+^CD8^−^ T cells from normal mice into Rag1^−/−^ mice leads to differentiation of DP IELs (194). Further studies reveal that in the context of TCR stimulation, TGF-β is the most important factor for the differentiation of DP IELs from peripheral CD4^+^CD8^−^ T cells (41, 196). The antigens should be mainly derived from the microbiome in the gut, as germ-free mice lack these DP IELs (195). Interestingly, TGF-β upregulates only CD8α mRNA, but not CD8β expression, in CD4^+^ T cells in culture (41). In vivo, after adoptive transfer TGFβRI-deficient CD4^+^CD8^−^ T cells failed to differentiate into DP IELs in Rag1^−/−^ mice (41). Although TGF-β induces both CD4^+^CD8αα^+^ and CD4^+^CD8αβ^+^ subsets in vitro (41), almost all DP IELs are CD4^+^CD8αα^+^ (195). This paradox was resolved by the findings that retinoic acid in the gut together with TGF-β preferentially induces CD4^+^CD8αα^+^ IELs (196). Mechanistically, TGF-β induces CD8α by suppressing ThPOK and upregulating RUNX3 expression in CD4^+^ T cells (41, 196), and it was suggested that the upregulation of RUNX3 occurs before ThPOK downregulation in CD4^+^ T cells (196). It has been observed that CD8α induction is in parallel with Foxp3 expression by TGF-β (41, 196), but only DP IELs, and not Foxp3^+^ Tregs, downregulate ThPOK expression (196). Interestingly, a recent study demonstrated that peripheral Foxp3^+^ Tregs, upon migration to the intestinal epithelium, lose their ThPOK and convert to DP IELs in a microbiota-dependent manner (197). However, the role of TGF-β in this conversion remains unknown. Functionally, DP IELs are considered to be cytotoxic T cells, as they express high levels of IFN-γ and granzyme B (196), although they also express IL-10 (194) and thus exhibit an immunoregulatory function in the gut. While TGF-β is required for differentiation of DP IELs, the role of TGF-β in their function remains largely unknown.

## 9. TGF-β IN INNATE LYMPHOID CELLS

ILCs do not express antigen-specific receptors, but they are important in protective immunity and regulation of homeostasis and inflammation (12). ILCs comprise three subsets. Group 1 ILCs (ILC1s) comprise the prototypical ILC1s and NK cells. ILC1s express NKp46, NK1.1, and transcription factor T-bet and produce IFN-γ. ILC2s express GATA-3 and produce IL-5 and IL-13. ILC3s produce IL-17A and IL-22 and depend on RORγt for differentiation. TGF-β has been reported to play roles in the differentiation of salivary gland ILC1s and the development of ILC2s (Figure 3).

### 9.1. TGF-β in ILC1s

TGF-β controls the differentiation of ILC1s in the salivary glands (198) and converts NK cells into ILC1-like cells in tumors and during viral infections (199, 200). Salivary gland ILC1s express a unique array of cell surface molecules, cytokines, and developmental transcription factors, namely surface CD103, CD49a, CD39, TRAIL, and CD69 (198). They also produce low amounts of IFN-γ and require T-bet and Eomes, but not Nfil3, for development. Conditional knockout mice with TGFβRII deletion specifically in ILC1s and NKp46^+^ cells (*Tgfbr2*^flox/flox^*Ncr1Cre*^+^) have fewer salivary gland ILCs, which lack their signature markers (198). Further studies reveal that TGF-β promotes salivary gland ILC1 differentiation by suppressing Eomes through Junk-dependent but Smad4-independent pathways. In addition, a recent report showed that TGF-β is also required for the maintenance of a granzyme C–expressing ILC1 subset in the salivary glands (201).

TGF-β may also convert NK cells into ILC1-like cells devoid of cytotoxic function (199, 200) in the tumor microenvironment. TGF-β induces conversion of NK cells (CD49a^−^CD49β^+^Eomes^+^) into intermediate ILC1 (CD49a^+^CD49b^+^Eomes^+^) populations and ILC1 (CD49a^+^CD49b^−^Eomes^int^) populations in the tumor microenvironment, which is mediated by Smad-independent pathways (199, 200). Actually, Smad4 inhibits TGF-β-mediated conversion of ILC1-like cells from NK cells: Smad4 deficiency in NK cells does not affect ILC1 differentiation, but Smad4^−/−^ NK cells acquire an ILC1-like gene signature and are unable to control tumor metastasis or viral infection (199). Importantly, TGF-β-mediated conversion of NK cells into ILC1-like cells also occurs in human NK cells. NK cells from a SMAD4-deficient patient affected by polyposis were also hyperresponsive to TGF-β (199). IL-15 may synergize with TGF-β in this cellular conversion in human NK cells (202). Analysis of downstream TGF-β signaling suggests that TAK1-mediated activation of p38 MAPK as the critical pathway driving the conversion and that IL-15 enhanced TGF-β-mediated conversion through Ras:RAC1 signaling as well as via the activation of MEK/ERK. However, human circulating NK cells treated with TGF-β show heterogeneity in their potential to adopt an ILC1-like phenotype, as indicated by the upregulation of CD9 and CD103 in only a subset of cells in culture. On the other hand, murine and human ILC1s secrete TGF-β, driving expansion of CD44v6^+^ epithelial crypts in the intestine (203).

### 9.2. TGF-β in ILC2s

In the bone marrow, TGF-β signaling programs the development of ILC2s, but surprisingly not ILC1s or ILC3s, from their progenitors (204). This is accomplished by TGF-β-mediated generation and maintenance of ILC2 progenitors. Mechanistically, TGF-β upregulates expression of the IL-33 receptor gene *Il1rl1* (encoding IL-1 receptor–like 1, also known as ST2) in ILC2 progenitors and common helper-like innate lymphoid progenitors (CHILPs), at least partially through a MEK-dependent and Smad3-independent pathway (204). In addition, TGF-β also maintains ST2 expression in mature ILC2s and protects their survival. Adoptive transfer of TβRII^ko^ ILC2s into Rag1^−/−^IL2rγ^−/−^ mice followed by induction of airway inflammation with house dust mites results in far fewer ILC2 effector cells and consequently substantially decreased lung inflammation compared to mice receiving wild-type ILC2s (204). Consistent with this, it was shown recently that TGF-β induces neuropilin-1 (Nrp1) in lung ILC2s and TGF-β-Nrp1 signaling enhances ILC2 function by upregulating ST2 expression (205). Moreover, TGF-β, whose activity is increased in systemic sclerosis, favored the expansion of KLRG1^−^ ILC2s while simultaneously decreasing their production of IL-10 in the skin of systemic sclerosis patients and increasing myofibroblast differentiation (206). Interestingly, TGF-β plays a role in promoting the conversion of c-Kit^−^ ILC2s into RORγt-expressing cells by inducing upregulation of IL23R, CCR6, and KIT mRNA in these cells (207).

### 9.3. TGF-β in ILC3s

Considering that ILC3s share some features of Th17 cells in that they produce IL-17 and IL-22, and that TGF-β is required for Th17 differentiation, it is somewhat surprising that no evidence thus far has been reported that TGF-β plays a role in the differentiation of ILC3s. ILC3s can be divided into two subsets based on the expression of the natural cytotoxicity receptor (NCR) NKp46 (encoded by *Ncr1*), namely NCR^+^ and NCR^−^ ILC3s (208, 209). Notch signaling is required for maintenance of NCR^+^ ILC3s and induces transition of NCR^−^ to NCR^+^ ILC3s (208). TGF-β, however, impairs the development of NCR^+^ ILC3s (209). A report suggests that TGF-β might initiate conversion of ILC3s toward regulatory ILCs (210) in the tumor microenvironment, but the significance of this conversion remains to be determined.

## 10. CONCLUSIONS AND PERSPECTIVE

Discovered as a transforming growth factor in nonimmune systems more than four decades ago (15), TGF-β has now been recognized as one of the most important cytokines regulating immune responses, especially T cell responses. Over the last 20 years, we have witnessed a conceptual revolution in how this ubiquitous and “nonspecific” cytokine acts, transforming from a simple suppressive performer that inhibits T cell activation and proliferation to a multifaceted conductor that orchestrates the development, homeostasis, differentiation, and death of almost all populations of T cells. Of particular note, the discoveries that TGF-β is critical for the differentiation of (*a*) CD4^+^ Tregs, Th17 cells, and Th9 cells; (*b*) CD8^+^ Trm cells, NKT cells, and TCRαβ^+^CD8αα^+^ and CD4^+^CD8αα^+^ IELs; and (*c*) salivary gland ILC1s and ILC2s have paved the way to a better understanding of T cell–mediated adoptive immunity and tolerance in immune homeostasis, as well as in the pathogenesis of autoimmunity, chronic inflammation, cancer, and infectious diseases. It is clear that the roles of TGF-β in the regulation of T cells not only are dependent on its signaling, but more importantly are also heavily influenced by signals from other molecules and cytokines in the microenvironment in which the T cells are located. Although we have learned a great deal about TGF-β function in T cells, many questions are yet to be resolved. For example, what is the missing link for the molecular and/or epigenetic mechanisms by which TGF-β signaling results in completely different Treg, Th17, and Th9 subpopulations in the presence of individual cytokines? How does TGF-β suppress CD8^+^ Teff cells but promote differentiation of Trm cells in the context of physiological and pathological conditions? What are the molecular mechanisms by which TGF-β is produced, processed, and activated in immune cells, particularly in T cells? What is the role of TGF-β during the cross talk between T cells and B cells or innate immune cells such as macrophages, neutrophils, and NK cells? Does TGF-β play a part in T cells and other immune cells during their interaction with microbiota? What is the function of TGF-β during the cross talk between T cells and tumor cells, stromal cells, or blood endothelial cells in the tumor microenvironment? How does TGF-β function in T cell–mediated antitumor immunotherapy in the context of checkpoint blockade and CAR (chimeric antigen receptor) T cells? How can we optimize and better manipulate TGF-β signaling to develop antigen-specific Tregs for the treatment of autoimmunity, and conversely how can we suppress tumor-specific Tregs for the treatment of cancer? What is the function of TGF-β in T cells for the development and pathogenesis of viral infections, such as HIV and SARS-CoV-2 infections, and can we manipulate TGF-β function to treat diseases caused by these infections? It will be important to understand the exact mechanisms by which TGF-β regulates different populations of T cells so as to understand the pathogenesis of related human diseases and develop more effective immunotherapies. We are hopeful that research into TGF-β regulation of immune responses including T cells over the next two decades will help revolutionize our understanding of and therapy for relevant human diseases.

## Supplementary Material

1

2

## References

[1] Sporn MB. 2006. The early history of TGF-beta, and a brief glimpse of its future. Cytokine Growth Factor Rev. 17:3–7 16290110 10.1016/j.cytogfr.2005.09.012

[2] Mosmann TR, Coffman RL. 1989. TH1 and TH2 cells: different patterns of lymphokine secretion lead to different functional properties. Annu. Rev. Immunol 7:145–73 2523712 10.1146/annurev.iy.07.040189.001045

[3] Szabo SJ, Kim ST, Costa GL, Zhang X, Fathman CG, Glimcher LH. 2000. A novel transcription factor, T-bet, directs Th1 lineage commitment. Cell 100:655–69 10761931 10.1016/s0092-8674(00)80702-3

[4] Zhu J, Paul WE. 2010. Peripheral CD4^+^ T-cell differentiation regulated by networks of cytokines and transcription factors. Immunol. Rev 238:247–62 20969597 10.1111/j.1600-065X.2010.00951.x PMC2975272

[5] Dong C. 2008. T_H_17 cells in development: an updated view of their molecular identity and genetic programming. Nat. Rev. Immunol 8:337–48 18408735 10.1038/nri2295

[6] Littman DR, Rudensky AY. 2010. Th17 and regulatory T cells in mediating and restraining inflammation. Cell 140:845–58 20303875 10.1016/j.cell.2010.02.021

[7] Kaplan MH, Hufford MM, Olson MR. 2015. The development and in vivo function of T helper 9 cells. Nat. Rev. Immunol 15:295–307 25848755 10.1038/nri3824 PMC4445728

[8] Sakaguchi S. 2000. Regulatory T cells: key controllers of immunologic self-tolerance. Cell 101:455–58 10850488 10.1016/s0092-8674(00)80856-9

[9] Crotty S. 2011. Follicular helper CD4 T cells (TFH). Annu. Rev. Immunol 29:621–63 21314428 10.1146/annurev-immunol-031210-101400

[10] Jameson SC, Masopust D. 2018. Understanding subset diversity in T cell memory. Immunity 48:214–26 29466754 10.1016/j.immuni.2018.02.010 PMC5863745

[11] Kok L, Masopust D, Schumacher TN. 2022. The precursors of CD8^+^ tissue resident memory T cells: from lymphoid organs to infected tissues. Nat. Rev. Immunol 22:283–93 34480118 10.1038/s41577-021-00590-3 PMC8415193

[12] Spits H, Artis D, Colonna M, Diefenbach A, Di Santo JP, 2013. Innate lymphoid cells—a proposal for uniform nomenclature. Nat. Rev. Immunol 13:145–49 23348417 10.1038/nri3365

[13] Cheroutre H, Lambolez F, Mucida D. 2011. The light and dark sides of intestinal intraepithelial lymphocytes. Nat. Rev. Immunol 11:445–56 21681197 10.1038/nri3007 PMC3140792

[14] Godfrey DI, MacDonald HR, Kronenberg M, Smyth MJ, Van Kaer L. 2004. NKT cells: What’s in a name? Nat. Rev. Immunol 4:231–37 15039760 10.1038/nri1309

[15] Roberts AB, Anzano MA, Lamb LC, Smith JM, Sporn MB. 1981. New class of transforming growth factors potentiated by epidermal growth factor: isolation from non-neoplastic tissues. PNAS 78:5339–43 6975480 10.1073/pnas.78.9.5339 PMC348740

[16] Chen W, Ten Dijke P. 2016. Immunoregulation by members of the TGFβ superfamily. Nat. Rev. Immunol 16:723–40 27885276 10.1038/nri.2016.112

[17] Massague J. 1996. TGFβ signaling: receptors, transducers, and Mad proteins. Cell 85:947–50 8674122 10.1016/s0092-8674(00)81296-9

[18] Bandyopadhyay B, Fan J, Guan S, Li Y, Chen M, 2006. A “traffic control” role for TGFβ3: orchestrating dermal and epidermal cell motility during wound healing. J. Cell Biol 172:1093–105 16549496 10.1083/jcb.200507111 PMC2063766

[19] Wu L, Siddiqui A, Morris DE, Cox DA, Roth SI, Mustoe TA. 1997. Transforming growth factor β3 (TGFβ3) accelerates wound healing without alteration of scar prominence: histologic and competitive reverse-transcription-polymerase chain reaction studies. Arch. Surg 132:753–60 9230861 10.1001/archsurg.1997.01430310067014

[20] Kulkarni AB, Huh CG, Becker D, Geiser A, Lyght M, 1993. Transforming growth factor beta 1 null mutation in mice causes excessive inflammatory response and early death. PNAS 90:770–74 8421714 10.1073/pnas.90.2.770 PMC45747

[21] Shull MM, Ormsby I, Kier AB, Pawlowski S, Diebold RJ, 1992. Targeted disruption of the mouse transforming growth factor-beta 1 gene results in multifocal inflammatory disease. Nature 359:693–99 1436033 10.1038/359693a0 PMC3889166

[22] Li MO, Sanjabi S, Flavell RA. 2006. Transforming growth factor-beta controls development, homeostasis, and tolerance of T cells by regulatory T cell-dependent and -independent mechanisms. Immunity 25:455–71 16973386 10.1016/j.immuni.2006.07.011

[23] Liu Y, Zhang P, Li J, Kulkarni AB, Perruche S, Chen W. 2008. A critical function for TGF-beta signaling in the development of natural CD4^+^CD25^+^Foxp3^+^ regulatory T cells. Nat. Immunol 9:632–40 18438410 10.1038/ni.1607

[24] Marie JC, Liggitt D, Rudensky AY. 2006. Cellular mechanisms of fatal early-onset autoimmunity in mice with the T cell-specific targeting of transforming growth factor-beta receptor. Immunity 25:441–54 16973387 10.1016/j.immuni.2006.07.012

[25] Takimoto T, Wakabayashi Y, Sekiya T, Inoue N, Morita R, 2010. Smad2 and Smad3 are redundantly essential for the TGF-beta-mediated regulation of regulatory T plasticity and Th1 development. J. Immunol 185:842–55 20548029 10.4049/jimmunol.0904100

[26] Derynck R, Zhang YE. 2003. Smad-dependent and Smad-independent pathways in TGF-beta family signalling. Nature 425:577–84 14534577 10.1038/nature02006

[27] Letterio JJ, Roberts AB. 1998. Regulation of immune responses by TGF-β. Annu. Rev. Immunol 16:137–61 9597127 10.1146/annurev.immunol.16.1.137

[28] Li MO, Wan YY, Sanjabi S, Robertson AK, Flavell RA. 2006. Transforming growth factor-β regulation of immune responses. Annu. Rev. Immunol 24:99–146 16551245 10.1146/annurev.immunol.24.021605.090737

[29] Massague J. 1996. TGFβ signaling: receptors, transducers, and Mad proteins. Cell 85:947–50 8674122 10.1016/s0092-8674(00)81296-9

[30] Rubtsov YP, Rudensky AY. 2007. TGFβ signalling in control of T-cell-mediated self-reactivity. Nat. Rev. Immunol 7:443–53 17525753 10.1038/nri2095

[31] Travis MA, Sheppard D. 2014. TGF-β activation and function in immunity. Annu. Rev. Immunol 32:51–82 24313777 10.1146/annurev-immunol-032713-120257 PMC4010192

[32] Moreau JM, Velegraki M, Bolyard C, Rosenblum MD, Li Z. 2022. Transforming growth factor-β1 in regulatory T cell biology. Sci. Immunol 7:eabi4613 10.1126/sciimmunol.abi4613 PMC10552796 35302863

[33] Shevach EM. 2017. Garp as a therapeutic target for modulation of T regulatory cell function. Expert Opin. Ther. Targets 21:191–200 28001437 10.1080/14728222.2017.1275568

[34] Pesu M, Watford WT, Wei L, Xu L, Fuss I, 2008. T-cell-expressed proprotein convertase furin is essential for maintenance of peripheral immune tolerance. Nature 455:246–50 18701887 10.1038/nature07210 PMC2758057

[35] Sorrentino A, Thakur N, Grimsby S, Marcusson A, von Bulow V, 2008. The type I TGF-beta receptor engages TRAF6 to activate TAK1 in a receptor kinase-independent manner. Nat. Cell Biol 10:1199–207 18758450 10.1038/ncb1780

[36] Yamashita M, Fatyol K, Jin C, Wang X, Liu Z, Zhang YE. 2008. TRAF6 mediates Smad-independent activation of JNK and p38 by TGF-beta. Mol. Cell 31:918–24 18922473 10.1016/j.molcel.2008.09.002 PMC2621323

[37] Chen W, Konkel JE. 2015. Development of thymic Foxp3^+^ regulatory T cells: TGF-beta matters. Eur. J. Immunol 45:958–65 25684698 10.1002/eji.201444999 PMC4428761

[38] Klein L, Robey EA, Hsieh CS. 2019. Central CD4^+^ T cell tolerance: deletion versus regulatory T cell differentiation. Nat. Rev. Immunol 19:7–18 30420705 10.1038/s41577-018-0083-6

[39] Konkel JE, Jin W, Abbatiello B, Grainger JR, Chen W. 2014. Thymocyte apoptosis drives the intrathymic generation of regulatory T cells. PNAS 111:E465–73 24474796 10.1073/pnas.1320319111 PMC3910656

[40] Ouyang W, Beckett O, Ma Q, Li MO. 2010. Transforming growth factor-beta signaling curbs thymic negative selection promoting regulatory T cell development. Immunity 32:642–53 20471291 10.1016/j.immuni.2010.04.012 PMC2880228

[41] Konkel JE, Maruyama T, Carpenter AC, Xiong Y, Zamarron BF, 2011. Control of the development of CD8αα^+^ intestinal intraepithelial lymphocytes by TGF-β. Nat. Immunol 12:312–19 21297643 10.1038/ni.1997 PMC3062738

[42] Etzensperger R, Kadakia T, Tai X, Alag A, Guinter TI, 2017. Identification of lineage-specifying cytokines that signal all CD8+-cytotoxic-lineage-fate ‘decisions’ in the thymus. Nat. Immunol 18:1218–27 28945245 10.1038/ni.3847 PMC5659273

[43] Ouyang W, Oh SA, Ma Q, Bivona MR, Zhu J, Li MO. 2013. TGF-beta cytokine signaling promotes CD8^+^ T cell development and low-affinity CD4^+^ T cell homeostasis by regulation of interleukin-7 receptor alpha expression. Immunity 39:335–46 23932572 10.1016/j.immuni.2013.07.016 PMC3801187

[44] Takada K, Jameson SC. 2009. Naive T cell homeostasis: from awareness of space to a sense of place. Nat. Rev. Immunol 9:823–32 19935802 10.1038/nri2657

[45] Jenkins MK. 1994. The ups and downs of T cell costimulation. Immunity 1:443–46 7534615 10.1016/1074-7613(94)90086-8

[46] Tu E, Chia CPZ, Chen W, Zhang D, Park SA, 2018. T cell receptor-regulated TGF-beta type I receptor expression determines T cell quiescence and activation. Immunity 48:745–59.e6 29669252 10.1016/j.immuni.2018.03.025 PMC5911925

[47] Flavell RA, Sanjabi S, Wrzesinski SH, Licona-Limon P. 2010. The polarization of immune cells in the tumour environment by TGFβ. Nat. Rev. Immunol 10:554–67 20616810 10.1038/nri2808 PMC3885992

[48] Gorelik L, Flavell RA. 2002. Transforming growth factor-beta in T-cell biology. Nat. Rev. Immunol 2:46–53 11905837 10.1038/nri704

[49] Harrington LE, Hatton RD, Mangan PR, Turner H, Murphy TL, 2005. Interleukin 17-producing CD4^+^ effector T cells develop via a lineage distinct from the T helper type 1 and 2 lineages. Nat. Immunol 6:1123–32 16200070 10.1038/ni1254

[50] Park H, Li Z, Yang XO, Chang SH, Nurieva R, 2005. A distinct lineage of CD4 T cells regulates tissue inflammation by producing interleukin 17. Nat. Immunol 6:1133–41 16200068 10.1038/ni1261 PMC1618871

[51] Korn T, Bettelli E, Oukka M, Kuchroo VK. 2009. IL-17 and Th17 Cells. Annu. Rev. Immunol 27:485–517 19132915 10.1146/annurev.immunol.021908.132710

[52] Cua DJ, Sherlock J, Chen Y, Murphy CA, Joyce B, 2003. Interleukin-23 rather than interleukin-12 is the critical cytokine for autoimmune inflammation of the brain. Nature 421:744–48 12610626 10.1038/nature01355

[53] Bettelli E, Carrier Y, Gao W, Korn T, Strom TB, 2006. Reciprocal developmental pathways for the generation of pathogenic effector T_H_17 and regulatory T cells. Nature 441:235–38 16648838 10.1038/nature04753

[54] Mangan PR, Harrington LE, O’Quinn DB, Helms WS, Bullard DC, 2006. Transforming growth factor-beta induces development of the T_H_17 lineage. Nature 441:231–34 16648837 10.1038/nature04754

[55] Veldhoen M, Hocking RJ, Atkins CJ, Locksley RM, Stockinger B. 2006. TGFβ in the context of an inflammatory cytokine milieu supports de novo differentiation of IL-17-producing T cells. Immunity 24:179–89 16473830 10.1016/j.immuni.2006.01.001

[56] Chen W, Jin W, Hardegen N, Lei KJ, Li L, 2003. Conversion of peripheral CD4^+^CD25^−^ naive T cells to CD4^+^CD25^+^ regulatory T cells by TGF-beta induction of transcription factor Foxp3. J. Exp. Med 198:1875–86 14676299 10.1084/jem.20030152 PMC2194145

[57] Zhou L, Littman DR. 2009. Transcriptional regulatory networks in Th17 cell differentiation. Curr. Opin. Immunol 21:146–52 19328669 10.1016/j.coi.2009.03.001 PMC2701391

[58] Yang XO, Panopoulos AD, Nurieva R, Chang SH, Wang D, 2007. STAT3 regulates cytokine-mediated generation of inflammatory helper T cells. J. Biol. Chem 282:9358–63 17277312 10.1074/jbc.C600321200

[59] Ghoreschi K, Laurence A, Yang XP, Hirahara K, O’Shea JJ. 2011. T helper 17 cell heterogeneity and pathogenicity in autoimmune disease. Trends Immunol. 32:395–401 21782512 10.1016/j.it.2011.06.007 PMC3163735

[60] Zhang S, Takaku M, Zou L, Gu AD, Chou WC, 2017. Reversing SKI-SMAD4-mediated suppression is essential for TH17 cell differentiation. Nature 551:105–9 29072299 10.1038/nature24283 PMC5743442

[61] Maruyama T, Li J, Vaque JP, Konkel JE, Wang W, 2011. Control of the differentiation of regulatory T cells and T_H_17 cells by the DNA-binding inhibitor Id3. Nat. Immunol 12:86–95 21131965 10.1038/ni.1965 PMC3140164

[62] Zhang F, Fuss IJ, Yang Z, Strober W. 2014. Transcription of RORγt in developing Th17 cells is regulated by E-proteins. Mucosal Immunol. 7:521–32 24064669 10.1038/mi.2013.69 PMC4381430

[63] Tanaka S, Jiang Y, Martinez GJ, Tanaka K, Yan X, 2018. Trim33 mediates the proinflammatory function of Th17 cells. J. Exp. Med 215:1853–68 29930104 10.1084/jem.20170779 PMC6028517

[64] Fadok VA, Bratton DL, Konowal A, Freed PW, Westcott JY, Henson PM. 1998. Macrophages that have ingested apoptotic cells in vitro inhibit proinflammatory cytokine production through autocrine/paracrine mechanisms involving TGF-beta, PGE2, and PAF. J. Clin. Investig 101:890–98 9466984 10.1172/JCI1112 PMC508637

[65] Kasagi S, Zhang P, Che L, Abbatiello B, Maruyama T, 2014. In vivo-generated antigen-specific regulatory T cells treat autoimmunity without compromising antibacterial immune response. Sci. Transl. Med 6:241ra78 10.1126/scitranslmed.3008895 24944193

[66] Perruche S, Zhang P, Liu Y, Saas P, Bluestone JA, Chen W. 2008. CD3-specific antibody-induced immune tolerance involves transforming growth factor-beta from phagocytes digesting apoptotic T cells. Nat. Med 14:528–35 18438416 10.1038/nm1749

[67] Esplugues E, Huber S, Gagliani N, Hauser AE, Town T, 2011. Control of T_H_17 cells occurs in the small intestine. Nature 475:514–18 21765430 10.1038/nature10228 PMC3148838

[68] Zhang D, Jin W, Wu R, Li J, Park SA, 2019. High glucose intake exacerbates autoimmunity through reactive-oxygen-species-mediated TGF-beta cytokine activation. Immunity 51:671–81.e5 31451397 10.1016/j.immuni.2019.08.001 PMC9811990

[69] Gutcher I, Donkor MK, Ma Q, Rudensky AY, Flavell RA, Li MO. 2011. Autocrine transforming growth factor-β1 promotes in vivo Th17 cell differentiation. Immunity 34:396–408 21435587 10.1016/j.immuni.2011.03.005 PMC3690311

[70] Diveu C, McGeachy MJ, Cua DJ. 2008. Cytokines that regulate autoimmunity. Curr. Opin. Immunol 20:663–68 18834938 10.1016/j.coi.2008.09.003

[71] Ahern PP, Schiering C, Buonocore S, McGeachy MJ, Cua DJ, 2010. Interleukin-23 drives intestinal inflammation through direct activity on T cells. Immunity 33:279–88 20732640 10.1016/j.immuni.2010.08.010 PMC3078329

[72] Ghoreschi K, Laurence A, Yang XP, Tato CM, McGeachy MJ, 2010. Generation of pathogenic T_H_17 cells in the absence of TGF-beta signalling. Nature 467:967–71 20962846 10.1038/nature09447 PMC3108066

[73] Lee Y, Awasthi A, Yosef N, Quintana FJ, Xiao S, 2012. Induction and molecular signature of pathogenic T_H_17 cells. Nat. Immunol 13:991–99 22961052 10.1038/ni.2416 PMC3459594

[74] Acosta-Rodriguez EV, Napolitani G, Lanzavecchia A, Sallusto F. 2007. Interleukins 1β and 6 but not transforming growth factor-β are essential for the differentiation of interleukin 17-producing human T helper cells. Nat. Immunol 8:942–49 17676045 10.1038/ni1496

[75] Chen Z, Tato CM, Muul L, Laurence A, O’Shea JJ. 2007. Distinct regulation of interleukin-17 in human T helper lymphocytes. Arthritis Rheum. 56:2936–46 17763419 10.1002/art.22866 PMC2323677

[76] van Beelen AJ, Zelinkova Z, Taanman-Kueter EW, Muller FJ, Hommes DW, 2007. Stimulation of the intracellular bacterial sensor NOD2 programs dendritic cells to promote interleukin-17 production in human memory T cells. Immunity 27:660–69 17919942 10.1016/j.immuni.2007.08.013

[77] Manel N, Unutmaz D, Littman DR. 2008. The differentiation of human T_H_-17 cells requires transforming growth factor-β and induction of the nuclear receptor RORγt. Nat. Immunol 9:641–49 18454151 10.1038/ni.1610 PMC2597394

[78] Lu Y, Hong S, Li H, Park J, Hong B, 2012. Th9 cells promote antitumor immune responses in vivo. J. Clin. Investig 122:4160–71 23064366 10.1172/JCI65459 PMC3484462

[79] Schmitt E, Germann T, Goedert S, Hoehn P, Huels C, 1994. IL-9 production of naive CD4^+^ T cells depends on IL-2, is synergistically enhanced by a combination of TGF-beta and IL-4, and is inhibited by IFN-gamma. J. Immunol 153:3989–96 7930607

[80] Dardalhon V, Awasthi A, Kwon H, Galileos G, Gao W, 2008. IL-4 inhibits TGF-beta-induced Foxp3^+^ T cells and, together with TGF-beta, generates IL-9^+^ IL-10^+^ Foxp3^−^ effector T cells. Nat. Immunol 9:1347–55 18997793 10.1038/ni.1677 PMC2999006

[81] Veldhoen M, Uyttenhove C, van Snick J, Helmby H, Westendorf A, 2008. Transforming growth factor-beta ‘reprograms’ the differentiation of T helper 2 cells and promotes an interleukin 9-producing subset. Nat. Immunol 9:1341–46 18931678 10.1038/ni.1659

[82] Beriou G, Bradshaw EM, Lozano E, Costantino CM, Hastings WD, 2010. TGF-beta induces IL-9 production from human Th17 cells. J. Immunol 185:46–54 20498357 10.4049/jimmunol.1000356 PMC2936106

[83] Uyttenhove C, Brombacher F, Van Snick J. 2010. TGF-beta interactions with IL-1 family members trigger IL-4-independent IL-9 production by mouse CD4^+^ T cells. Eur. J. Immunol 40:2230–35 20540113 10.1002/eji.200940281

[84] Wong MT, Ye JJ, Alonso MN, Landrigan A, Cheung RK, 2010. Regulation of human Th9 differentiation by type I interferons and IL-21. Immunol. Cell Biol 88:624–31 20421880 10.1038/icb.2010.53 PMC3090036

[85] Angkasekwinai P, Chang SH, Thapa M, Watarai H, Dong C. 2010. Regulation of IL-9 expression by IL-25 signaling. Nat. Immunol 11:250–56 20154671 10.1038/ni.1846 PMC2827302

[86] Nakatsukasa H, Zhang D, Maruyama T, Chen H, Cui K, 2015. The DNA-binding inhibitor Id3 regulates IL-9 production in CD4^+^ T cells. Nat. Immunol 16:1077–84 26322481 10.1038/ni.3252 PMC5935106

[87] Xiao X, Balasubramanian S, Liu W, Chu X, Wang H, 2012. OX40 signaling favors the induction of T_H_9 cells and airway inflammation. Nat. Immunol 13:981–90 22842344 10.1038/ni.2390 PMC3806044

[88] Kaplan MH. 2017. The transcription factor network in Th9 cells. Semin. Immunopathol 39:11–20 27837254 10.1007/s00281-016-0600-2 PMC5225158

[89] Elyaman W, Bassil R, Bradshaw EM, Orent W, Lahoud Y, 2012. Notch receptors and Smad3 signaling cooperate in the induction of interleukin-9-producing T cells. Immunity 36:623–34 22503540 10.1016/j.immuni.2012.01.020 PMC3572366

[90] Tamiya T, Ichiyama K, Kotani H, Fukaya T, Sekiya T, 2013. Smad2/3 and IRF4 play a cooperative role in IL-9-producing T cell induction. J. Immunol 191:2360–71 23913959 10.4049/jimmunol.1301276

[91] Wang A, Pan D, Lee YH, Martinez GJ, Feng XH, Dong C. 2013. Cutting edge: Smad2 and Smad4 regulate TGF-β-mediated *Il9* gene expression via EZH2 displacement. J. Immunol 191:4908–12 24108699 10.4049/jimmunol.1300433 PMC3842015

[92] Wilhelm C, Turner JE, Van Snick J, Stockinger B. 2012. The many lives of IL-9: a question of survival? Nat. Immunol 13:637–41 22713829 10.1038/ni.2303

[93] Fontenot JD, Gavin MA, Rudensky AY. 2003. Foxp3 programs the development and function of CD4^+^CD25^+^ regulatory T cells. Nat. Immunol 4:330–36 12612578 10.1038/ni904

[94] Hori S, Nomura T, Sakaguchi S. 2003. Control of regulatory T cell development by the transcription factor Foxp3. Science 299:1057–61 12522256 10.1126/science.1079490

[95] Khattri R, Cox T, Yasayko SA, Ramsdell F. 2003. An essential role for Scurfin in CD4^+^CD25^+^ T regulatory cells. Nat. Immunol 4:337–42 12612581 10.1038/ni909

[96] Kretschmer K, Apostolou I, Hawiger D, Khazaie K, Nussenzweig MC, von Boehmer H. 2005. Inducing and expanding regulatory T cell populations by foreign antigen. Nat. Immunol 6:1219–27 16244650 10.1038/ni1265

[97] Sakaguchi S, Sakaguchi N, Asano M, Itoh M, Toda M. 1995. Immunologic self-tolerance maintained by activated T cells expressing IL-2 receptor alpha-chains (CD25): Breakdown of a single mechanism of self-tolerance causes various autoimmune diseases. J. Immunol 155:1151–64 7636184

[98] Shevach EM. 2009. Mechanisms of foxp3^+^ T regulatory cell-mediated suppression. Immunity 30:636–45 19464986 10.1016/j.immuni.2009.04.010

[99] Chen WJ, Frank M, Jin WW, Lei KJ, Hardegen N, Wahl SM. 2001. TGF-β induces anergic/suppressor CD4^+^ CD25^+^ CTLA-4^+^ T cells. J. Leuk. Biol 2001(Suppl.):102 (Abstr.)

[100] Yamagiwa S, Gray JD, Hashimoto S, Horwitz DA. 2001. A role for TGF-beta in the generation and expansion of CD4^+^CD25^+^ regulatory T cells from human peripheral blood. J. Immunol 166:7282–89 11390478 10.4049/jimmunol.166.12.7282

[101] Cobbold SP, Castejon R, Adams E, Zelenika D, Graca L, 2004. Induction of *foxP3*^+^ regulatory T cells in the periphery of T cell receptor transgenic mice tolerized to transplants. J. Immunol 172:6003–10 15128783 10.4049/jimmunol.172.10.6003

[102] Fantini MC, Becker C, Monteleone G, Pallone F, Galle PR, Neurath MF. 2004. Cutting edge: TGF-beta induces a regulatory phenotype in CD4^+^CD25^−^ T cells through Foxp3 induction and down-regulation of Smad7. J. Immunol 172:5149–53 15100250 10.4049/jimmunol.172.9.5149

[103] Fu S, Zhang N, Yopp AC, Chen D, Mao M, 2004. TGF-β induces Foxp3 + T-regulatory cells from CD4 + CD25 − precursors. Am. J. Transplant 4:1614–27 15367216 10.1111/j.1600-6143.2004.00566.x

[104] Rao PE, Petrone AL, Ponath PD. 2005. Differentiation and expansion of T cells with regulatory function from human peripheral lymphocytes by stimulation in the presence of TGF-β. J. Immunol 174:1446–55 15661903 10.4049/jimmunol.174.3.1446

[105] Zheng SG, Wang JH, Gray JD, Soucier H, Horwitz DA. 2004. Natural and induced CD4^+^CD25^+^ cells educate CD4^+^CD25^−^ cells to develop suppressive activity: the role of IL-2, TGF-beta, and IL-10. J. Immunol 172:5213–21 15100259 10.4049/jimmunol.172.9.5213

[106] Benson MJ, Pino-Lagos K, Rosemblatt M, Noelle RJ. 2007. All-trans retinoic acid mediates enhanced T reg cell growth, differentiation, and gut homing in the face of high levels of co-stimulation. J. Exp. Med 204:1765–74 17620363 10.1084/jem.20070719 PMC2118687

[107] Coombes JL, Siddiqui KR, Arancibia-Carcamo CV, Hall J, Sun CM, 2007. A functionally specialized population of mucosal CD103^+^ DCs induces Foxp3^+^ regulatory T cells via a TGF-β− and retinoic acid–dependent mechanism. J. Exp. Med 204:1757–64 17620361 10.1084/jem.20070590 PMC2118683

[108] Josefowicz SZ, Niec RE, Kim HY, Treuting P, Chinen T, 2012. Extrathymically generated regulatory T cells control mucosal T_H_2 inflammation. Nature 482:395–99 22318520 10.1038/nature10772 PMC3485072

[109] Sun CM, Hall JA, Blank RB, Bouladoux N, Oukka M, 2007. Small intestine lamina propria dendritic cells promote de novo generation of Foxp3 T reg cells via retinoic acid. J. Exp. Med 204:1775–85 17620362 10.1084/jem.20070602 PMC2118682

[110] Mucida D, Park Y, Kim G, Turovskaya O, Scott I, 2007. Reciprocal T_H_17 and regulatory T cell differentiation mediated by retinoic acid. Science 317:256–60 17569825 10.1126/science.1145697

[111] Floess S, Freyer J, Siewert C, Baron U, Olek S, 2007. Epigenetic control of the *foxp3* locus in regulatory T cells. PLOS Biol. 5:e38 17298177 10.1371/journal.pbio.0050038 PMC1783672

[112] Yue X, Trifari S, Aijo T, Tsagaratou A, Pastor WA, 2016. Control of Foxp3 stability through modulation of TET activity. J. Exp. Med 213:377–97 26903244 10.1084/jem.20151438 PMC4813667

[113] Yue X, Samaniego-Castruita D, Gonzalez-Avalos E, Li X, Barwick BG, Rao A. 2021. Whole-genome analysis of TET dioxygenase function in regulatory T cells. EMBO Rep. 22:e52716 10.15252/embr.202152716 PMC8339674 34288360

[114] Chen W, Frank ME, Jin W, Wahl SM. 2001. TGF-beta released by apoptotic T cells contributes to an immunosuppressive milieu. Immunity 14:715–25 11420042 10.1016/s1074-7613(01)00147-9

[115] Chen X, Yang X, Yuan P, Jin R, Bao L, 2021. Modular immune-homeostatic microparticles promote immune tolerance in mouse autoimmune models. Sci. Transl. Med 13:eaaw9668 10.1126/scitranslmed.aaw9668 PMC12230986 33692135

[116] Chen Z, Zhang T, Kam HT, Qiao D, Jin W, 2021. Induction of antigen-specific Treg cells in treating autoimmune uveitis via bystander suppressive pathways without compromising anti-tumor immunity. eBioMedicine 70:103496 10.1016/j.ebiom.2021.103496 PMC8318874 34280776

[117] Xu J, Liu O, Wang D, Wang F, Zhang D, 2022. In vivo generating SSA/Ro-antigen specific regulatory T cells improves experimental Sjögren’s syndrome in mice. Arthritis Rheumatol. 74(10):1699–705 35606923 10.1002/art.42244 PMC9811988

[118] Zhang D, Chia C, Jiao X, Jin W, Kasagi S, 2017. D-Mannose induces regulatory T cells and suppresses immunopathology. Nat. Med 23:1036–45 28759052 10.1038/nm.4375 PMC12180587

[119] Sakaguchi S, Miyara M, Costantino CM, Hafler DA. 2010. FOXP3^+^ regulatory T cells in the human immune system. Nat. Rev. Immunol 10:490–500 20559327 10.1038/nri2785

[120] Amarnath S, Dong L, Li J, Wu Y, Chen W. 2007. Endogenous TGF-beta activation by reactive oxygen species is key to Foxp3 induction in TCR-stimulated and HIV-1-infected human CD4^+^CD25^−^ T cells. Retrovirology 4:57 17688698 10.1186/1742-4690-4-57 PMC2096626

[121] Tran DQ, Ramsey H, Shevach EM. 2007. Induction of FOXP3 expression in naive human CD4^+^FOXP3 T cells by T-cell receptor stimulation is transforming growth factor-beta dependent but does not confer a regulatory phenotype. Blood 110:2983–90 17644734 10.1182/blood-2007-06-094656 PMC2018674

[122] Chen W, Jin W, Tian H, Sicurello P, Frank M, 2001. Requirement for transforming growth factor β1 in controlling T cell apoptosis. J. Exp. Med 194:439–53 11514601 10.1084/jem.194.4.439 PMC2193497

[123] Kawakami R, Kitagawa Y, Chen KY, Arai M, Ohara D, 2021. Distinct Foxp3 enhancer elements coordinate development, maintenance, and function of regulatory T cells. Immunity 54:947–61.e8 33930308 10.1016/j.immuni.2021.04.005

[124] Kim HP, Leonard WJ. 2007. CREB/ATF-dependent T cell receptor-induced FoxP3 gene expression: a role for DNA methylation. J. Exp. Med 204:1543–51 17591856 10.1084/jem.20070109 PMC2118651

[125] Kitagawa Y, Ohkura N, Kidani Y, Vandenbon A, Hirota K, 2017. Guidance of regulatory T cell development by Satb1-dependent super-enhancer establishment. Nat. Immunol 18:173–83 27992401 10.1038/ni.3646 PMC5582804

[126] Placek K, Hu G, Cui K, Zhang D, Ding Y, 2017. MLL4 prepares the enhancer landscape for Foxp3 induction via chromatin looping. Nat. Immunol 18:1035–45 28759003 10.1038/ni.3812 PMC5836551

[127] Ruan Q, Kameswaran V, Tone Y, Li L, Liou HC, 2009. Development of Foxp3^+^ regulatory T cells is driven by the c-Rel enhanceosome. Immunity 31:932–40 20064450 10.1016/j.immuni.2009.10.006 PMC2807990

[128] Tone Y, Furuuchi K, Kojima Y, Tykocinski ML, Greene MI, Tone M. 2008. Smad3 and NFAT cooperate to induce Foxp3 expression through its enhancer. Nat. Immunol 9:194–202 18157133 10.1038/ni1549

[129] Zheng Y, Josefowicz S, Chaudhry A, Peng XP, Forbush K, Rudensky AY. 2010. Role of conserved noncoding DNA elements in the Foxp3 gene in regulatory T-cell fate. Nature 463:808–12 20072126 10.1038/nature08750 PMC2884187

[130] Dikiy S, Li J, Bai L, Jiang M, Janke L, 2021. A distal Foxp3 enhancer enables interleukin-2 dependent thymic Treg cell lineage commitment for robust immune tolerance. Immunity 54:931–46.e11 33838102 10.1016/j.immuni.2021.03.020 PMC8317508

[131] Chen W, Wahl SM. 2003. TGF-beta: the missing link in CD4^+^CD25^+^ regulatory T cell-mediated immunosuppression. Cytokine Growth Factor. Rev 14:85–89 12651220 10.1016/s1359-6101(03)00003-0

[132] Nakamura K, Kitani A, Strober W. 2001. Cell contact-dependent immunosuppression by CD4^+^CD25^+^ regulatory T cells is mediated by cell surface-bound transforming growth factor beta. J. Exp. Med 194:629–44 11535631 10.1084/jem.194.5.629 PMC2195935

[133] Stockis J, Colau D, Coulie PG, Lucas S. 2009. Membrane protein GARP is a receptor for latent TGF-beta on the surface of activated human Treg. Eur. J. Immunol 39:3315–22 19750484 10.1002/eji.200939684

[134] Azhar M, Yin M, Bommireddy R, Duffy JJ, Yang J, 2009. Generation of mice with a conditional allele for transforming growth factor beta 1 gene. Genesis 47:423–31 19415629 10.1002/dvg.20516 PMC2766615

[135] Choi G, Kim BS, Chang JH, Chung Y. 2021. Defining the role of transforming growth factor β1 in Foxp3^+^ T regulatory cells. Immunity 54:393–94 33691125 10.1016/j.immuni.2021.02.008

[136] Turner JA, Stephen-Victor E, Wang S, Rivas MN, Abdel-Gadir A, 2020. Regulatory T cell-derived TGF-β1 controls multiple checkpoints governing allergy and autoimmunity. Immunity 53:1202–14.e6 33086036 10.1016/j.immuni.2020.10.002 PMC7744401

[137] Velegraki M, Salem M, Ansa-Addo EA, Wu BX, Li Z. 2021. Autocrine transforming growth factor β1 in regulatory T cell biology—gone but not missed. Immunity 54:395–96 33691126 10.1016/j.immuni.2021.02.007

[138] Wang R, Zhu J, Dong X, Shi M, Lu C, Springer TA. 2012. GARP regulates the bioavailability and activation of TGFβ. Mol. Biol. Cell 23:1129–39 22278742 10.1091/mbc.E11-12-1018 PMC3302739

[139] Worthington JJ, Kelly A, Smedley C, Bauche D, Campbell S, 2015. Integrin αvβ8-mediated TGF-β activation by effector regulatory T cells is essential for suppression of T-cell-mediated inflammation. Immunity 42:903–15 25979421 10.1016/j.immuni.2015.04.012 PMC4448149

[140] Travis MA, Reizis B, Melton AC, Masteller E, Tang Q, 2007. Loss of integrin α_v_β_8_ on dendritic cells causes autoimmunity and colitis in mice. Nature 449:361–65 17694047 10.1038/nature06110 PMC2670239

[141] Metelli A, Wu BX, Riesenberg B, Guglietta S, Huck JD, 2020. Thrombin contributes to cancer immune evasion via proteolysis of platelet-bound GARP to activate LTGF-β. Sci. Transl. Med 12:eaay4860 10.1126/scitranslmed.aay4860 PMC7814995 31915300

[142] Koch MA, Tucker-Heard G, Perdue NR, Killebrew JR, Urdahl KB, Campbell DJ. 2009. The transcription factor T-bet controls regulatory T cell homeostasis and function during type 1 inflammation. Nat. Immunol 10:595–602 19412181 10.1038/ni.1731 PMC2712126

[143] Chaudhry A, Rudra D, Treuting P, Samstein RM, Liang Y, 2009. CD4^+^ regulatory T cells control T_H_17 responses in a Stat3-dependent manner. Science 326:986–91 19797626 10.1126/science.1172702 PMC4408196

[144] Konkel JE, Zhang D, Zanvit P, Chia C, Zangarle-Murray T, 2017. Transforming growth factor-beta signaling in regulatory T cells controls T helper-17 cells and tissue-specific immune responses. Immunity 46:660–74 28423340 10.1016/j.immuni.2017.03.015 PMC12230991

[145] Yang K, Kallies A. 2021. Tissue-specific differentiation of CD8^+^ resident memory T cells. Trends Immunol. 42:876–90 34531111 10.1016/j.it.2021.08.002

[146] Mishra S, Liao W, Liu Y, Yang M, Ma C, 2021. TGF-β and Eomes control the homeostasis of CD8^+^ regulatory T cells. J. Exp. Med 218:e20200030 10.1084/jem.20200030 PMC7527976 32991667

[147] McCarron MJ, Marie JC. 2014. TGF-beta prevents T follicular helper cell accumulation and B cell autoreactivity. J. Clin. Investig 124:4375–86 25157822 10.1172/JCI76179 PMC4191003

[148] Kim YJ, Stringfield TM, Chen Y, Broxmeyer HE. 2005. Modulation of cord blood CD8+ T-cell effector differentiation by TGF-β1 and 4–1BB costimulation. Blood 105:274–81 15353478 10.1182/blood-2003-12-4343

[149] McKarns SC, Schwartz RH. 2005. Distinct effects of TGF-beta 1 on CD4^+^ and CD8^+^ T cell survival, division, and IL-2 production: a role for T cell intrinsic Smad3. J. Immunol 174:2071–83 15699137 10.4049/jimmunol.174.4.2071

[150] Thomas DA, Massague J. 2005. TGF-beta directly targets cytotoxic T cell functions during tumor evasion of immune surveillance. Cancer Cell 8:369–80 16286245 10.1016/j.ccr.2005.10.012

[151] Budhu S, Schaer DA, Li Y, Toledo-Crow R, Panageas K, 2017. Blockade of surface-bound TGF-β on regulatory T cells abrogates suppression of effector T cell function in the tumor microenvironment. Sci. Signal 10:eaak9702 10.1126/scisignal.aak9702 PMC5851440 28851824

[152] Mariathasan S, Turley SJ, Nickles D, Castiglioni A, Yuen K, 2018. TGFβ attenuates tumour response to PD-L1 blockade by contributing to exclusion of T cells. Nature 554:544–48 29443960 10.1038/nature25501 PMC6028240

[153] Tauriello DVF, Palomo-Ponce S, Stork D, Berenguer-Llergo A, Badia-Ramentol J, 2018. TGFβ drives immune evasion in genetically reconstituted colon cancer metastasis. Nature 554:538–43 29443964 10.1038/nature25492

[154] Park BV, Freeman ZT, Ghasemzadeh A, Chattergoon MA, Rutebemberwa A, 2016. TGFβ1-media3ted SMAD3 enhances PD-1 expression on antigen-specific T cells in cancer. Cancer Discov. 6:366–81 10.1158/2159-8290.CD-15-1347 PMC5295786 27683557

[155] Alvarez M, Bouchlaka MN, Sckisel GD, Sungur CM, Chen M, Murphy WJ. 2014. Increased antitumor effects using IL-2 with anti-TGF-β reveals competition between mouse NK and CD8 T cells. J. Immunol 193:1709–16 25000978 10.4049/jimmunol.1400034 PMC4241855

[156] Bellavance EC, Kohlhapp FJ, Zloza A, O’Sullivan JA, McCracken J, 2011. Development of tumor-infiltrating CD8^+^ T cell memory precursor effector cells and antimelanoma memory responses are the result of vaccination and TGF-beta blockade during the perioperative period of tumor resection. J. Immunol 186:3309–16 21289306 10.4049/jimmunol.1002549 PMC3048906

[157] Sanjabi S, Mosaheb MM, Flavell RA. 2009. Opposing effects of TGF-beta and IL-15 cytokines control the number of short-lived effector CD8^+^ T cells. Immunity 31:131–44 19604492 10.1016/j.immuni.2009.04.020 PMC2765785

[158] Gate D, Danielpour M, Rodriguez J Jr., Kim GB, Levy R, 2014. T-cell TGF-beta signaling abrogation restricts medulloblastoma progression. PNAS 111:E3458–66 25082897 10.1073/pnas.1412489111 PMC4143044

[159] Wallberg M, Wong FS, Green EA. 2011. An islet-specific pulse of TGF-beta abrogates CTL function and promotes beta cell survival independent of Foxp3^+^ T cells. J. Immunol 186:2543–51 21217013 10.4049/jimmunol.1002098

[160] Zhang N, Bevan MJ. 2012. TGF-beta signaling to T cells inhibits autoimmunity during lymphopenia-driven proliferation. Nat. Immunol 13:667–73 22634866 10.1038/ni.2319 PMC3380154

[161] Bromley SK, Akbaba H, Mani V, Mora-Buch R, Chasse AY, 2020. CD49a regulates cutaneous resident memory CD8^+^ T cell persistence and response. Cell Rep. 32:108085 10.1016/j.celrep.2020.108085 PMC7520726 32877667

[162] Mackay LK, Rahimpour A, Ma JZ, Collins N, Stock AT, 2013. The developmental pathway for CD103^+^CD8^+^ tissue-resident memory T cells of skin. Nat. Immunol 14:1294–301 24162776 10.1038/ni.2744

[163] Sheridan BS, Pham QM, Lee YT, Cauley LS, Puddington L, Lefrancois L. 2014. Oral infection drives a distinct population of intestinal resident memory CD8^+^ T cells with enhanced protective function. Immunity 40:747–57 24792910 10.1016/j.immuni.2014.03.007 PMC4045016

[164] Borges da Silva H, Peng C, Wang H, Wanhainen KM, Ma C, 2020. Sensing of ATP via the purinergic receptor P2RX7 promotes CD8^+^ Trm cell generation by enhancing their sensitivity to the cytokine TGF-β. Immunity 53:158–71.e6 32640257 10.1016/j.immuni.2020.06.010 PMC8026201

[165] Laidlaw BJ, Zhang N, Marshall HD, Staron MM, Guan T, 2014. CD4^+^ T cell help guides formation of CD103^+^ lung-resident memory CD8^+^ T cells during influenza viral infection. Immunity 41:633–45 25308332 10.1016/j.immuni.2014.09.007 PMC4324721

[166] Wu J, Madi A, Mieg A, Hotz-Wagenblatt A, Weisshaar N, 2020. T cell factor 1 suppresses CD103+ lung tissue-resident memory T cell development. Cell Rep. 31:107484 10.1016/j.celrep.2020.03.048 32268106

[167] Qiu Z, Chu TH, Sheridan BS. 2021. TGF-β: many paths to CD103^+^ CD8 T cell residency. Cells 10:989 33922441 10.3390/cells10050989 PMC8145941

[168] Loi JK, Alexandre YO, Senthil K, Schienstock D, Sandford S, 2022. Corneal tissue-resident memory T cells form a unique immune compartment at the ocular surface. Cell Rep. 39:110852 10.1016/j.celrep.2022.110852 35613584

[169] Ray SJ, Franki SN, Pierce RH, Dimitrova S, Koteliansky V, 2004. The collagen binding α1β1 integrin VLA-1 regulates CD8 T cell-mediated immune protection against heterologous influenza infection. Immunity 20:167–79 14975239 10.1016/s1074-7613(04)00021-4

[170] Skon CN, Lee JY, Anderson KG, Masopust D, Hogquist KA, Jameson SC. 2013. Transcriptional downregulation of *S1pr1* is required for the establishment of resident memory CD8^+^ T cells. Nat. Immunol 14:1285–93 24162775 10.1038/ni.2745 PMC3844557

[171] Mani V, Bromley SK, Aijo T, Mora-Buch R, Carrizosa E, 2019. Migratory DCs activate TGF-β to precondition naïve CD8^+^ T cells for tissue-resident memory fate. Science 366:eaav5728 10.1126/science.aav5728 PMC6939608 31601741

[172] Ferreira C, Barros L, Baptista M, Blankenhaus B, Barros A, 2020. Type 1 Treg cells promote the generation of CD8^+^ tissue-resident memory T cells. Nat. Immunol 21:766–76 32424367 10.1038/s41590-020-0674-9

[173] Malenica I, Adam J, Corgnac S, Mezquita L, Auclin E, 2021. Integrin-α_V_-mediated activation of TGF-β regulates anti-tumour CD8 T cell immunity and response to PD-1 blockade. Nat. Commun 12:5209 34471106 10.1038/s41467-021-25322-y PMC8410945

[174] Kallies A, Zehn D, Utzschneider DT. 2020. Precursor exhausted T cells: key to successful immunotherapy? Nat. Rev. Immunol 20:128–36 31591533 10.1038/s41577-019-0223-7

[175] Chatterjee S, Chatterjee A, Jana S, Dey S, Roy H, 2021. Transforming growth factor beta orchestrates PD-L1 enrichment in tumor-derived exosomes and mediates CD8 T-cell dysfunction regulating early phosphorylation of TCR signalome in breast cancer. Carcinogenesis 42:38–47 32832992 10.1093/carcin/bgaa092

[176] Giordano M, Henin C, Maurizio J, Imbratta C, Bourdely P, 2015. Molecular profiling of CD8 T cells in autochthonous melanoma identifies Maf as driver of exhaustion. EMBO J. 34:2042–58 26139534 10.15252/embj.201490786 PMC4551351

[177] Smith CJ, Snyder CM. 2021. Inhibitory molecules PD-1, CD73 and CD39 are expressed by CD8^+^ T cells in a tissue-dependent manner and can inhibit T cell responses to stimulation. Front. Immunol 12:704862 10.3389/fimmu.2021.704862 PMC8320728 34335618

[178] Stephen TL, Rutkowski MR, Allegrezza MJ, Perales-Puchalt A, Tesone AJ, 2014. Transforming growth factor beta-mediated suppression of antitumor T cells requires FoxP1 transcription factor expression. Immunity 41:427–39 25238097 10.1016/j.immuni.2014.08.012 PMC4174366

[179] Tinoco R, Alcalde V, Yang Y, Sauer K, Zuniga EI. 2009. Cell-intrinsic transforming growth factor-beta signaling mediates virus-specific CD8^+^ T cell deletion and viral persistence in vivo. Immunity 31:145–57 19604493 10.1016/j.immuni.2009.06.015 PMC3039716

[180] Gabriel SS, Tsui C, Chisanga D, Weber F, Llano-Leon M, 2021. Transforming growth factor-beta-regulated mTOR activity preserves cellular metabolism to maintain long-term T cell responses in chronic infection. Immunity 54:1698–714.e5 34233154 10.1016/j.immuni.2021.06.007

[181] Pomie C, Menager-Marcq I, van Meerwijk JP. 2008. Murine CD8^+^ regulatory T lymphocytes: the new era. Hum. Immunol 69:708–14 18817827 10.1016/j.humimm.2008.08.288

[182] Kim HJ, Verbinnen B, Tang X, Lu L, Cantor H. 2010. Inhibition of follicular T-helper cells by CD8^+^ regulatory T cells is essential for self tolerance. Nature 467:328–32 20844537 10.1038/nature09370 PMC3395240

[183] Li J, Zaslavsky M, Su Y, Guo J, Sikora MJ, 2022. KIR^+^CD8^+^ T cells suppress pathogenic T cells and are active in autoimmune diseases and COVID-19. Science 376:eabi9591 10.1126/science.abi9591 PMC8995031 35258337

[184] Du J, Yang H, Zhang D, Wang J, Guo H, 2010. Structural basis for the blockage of IL-2 signaling by therapeutic antibody basiliximab. J. Immunol 184:1361–68 20032294 10.4049/jimmunol.0903178

[185] Hu B, Jin C, Zeng X, Resch JM, Jedrychowski MP, 2020. γδ T cells and adipocyte IL-17RC control fat innervation and thermogenesis. Nature 578:610–14 32076265 10.1038/s41586-020-2028-z PMC7055484

[186] Peters C, Hasler R, Wesch D, Kabelitz D. 2016. Human Vδ2 T cells are a major source of interleukin-9. PNAS 113:12520–25 27791087 10.1073/pnas.1607136113 PMC5098669

[187] Bendelac A, Savage PB, Teyton L. 2007. The biology of NKT cells. Annu. Rev. Immunol 25:297–336 17150027 10.1146/annurev.immunol.25.022106.141711

[188] Chuang YH, Lian ZX, Yang GX, Shu SA, Moritoki Y, 2008. Natural killer T cells exacerbate liver injury in a transforming growth factor beta receptor II dominant-negative mouse model of primary biliary cirrhosis. Hepatology 47:571–80 18098320 10.1002/hep.22052

[189] Monteiro M, Agua-Doce A, Almeida CF, Fonseca-Pereira D, Veiga-Fernandes H, Graca L. 2015. IL-9 expression by invariant NKT cells is not imprinted during thymic development. J. Immunol 195:3463–71 26297763 10.4049/jimmunol.1403170

[190] McDonald BD, Jabri B, Bendelac A. 2018. Diverse developmental pathways of intestinal intraepithelial lymphocytes. Nat. Rev. Immunol 18:514–25 29717233 10.1038/s41577-018-0013-7 PMC6063796

[191] Suzuki R, Nakao A, Kanamaru Y, Okumura K, Ogawa H, Ra C. 2002. Localization of intestinal intraepithelial T lymphocytes involves regulation of αEβ7 expression by transforming growth factor-β. Int. Immunol 14:339–45 11934870 10.1093/intimm/14.4.339

[192] Lim SP, Leung E, Krissansen GW. 1998. The β7 integrin gene (Itgb-7) promoter is responsive to TGF-β1: defining control regions. Immunogenetics 48:184–95 9683663 10.1007/s002510050422

[193] Verstichel G, Vermijlen D, Martens L, Goetgeluk G, Brouwer M, 2017. The checkpoint for agonist selection precedes conventional selection in human thymus. Sci. Immunol 2:eaah4232 10.1126/sciimmunol.aah4232 PMC5569900 28783686

[194] Das G, Augustine MM, Das J, Bottomly K, Ray P, Ray A. 2003. An important regulatory role for CD4^+^CD8αα T cells in the intestinal epithelial layer in the prevention of inflammatory bowel disease. PNAS 100:5324–29 12695566 10.1073/pnas.0831037100 PMC154344

[195] Mucida D, Husain MM, Muroi S, van Wijk F, Shinnakasu R, 2013. Transcriptional reprogramming of mature CD4^+^ helper T cells generates distinct MHC class II-restricted cytotoxic T lymphocytes. Nat. Immunol 14:281–89 23334788 10.1038/ni.2523 PMC3581083

[196] Reis BS, Rogoz A, Costa-Pinto FA, Taniuchi I, Mucida D. 2013. Mutual expression of the transcription factors Runx3 and ThPOK regulates intestinal CD4^+^ T cell immunity. Nat. Immunol 14:271–80 23334789 10.1038/ni.2518 PMC3804366

[197] Sujino T, London M, Hoytema van Konijnenburg DP, Rendon T, Buch T, 2016. Tissue adaptation of regulatory and intraepithelial CD4^+^ T cells controls gut inflammation. Science 352:1581–86 27256884 10.1126/science.aaf3892 PMC4968079

[198] Cortez VS, Cervantes-Barragan L, Robinette ML, Bando JK, Wang Y, 2016. Transforming growth factor-β signaling guides the differentiation of innate lymphoid cells in salivary glands. Immunity 44:1127–39 27156386 10.1016/j.immuni.2016.03.007 PMC5114145

[199] Cortez VS, Ulland TK, Cervantes-Barragan L, Bando JK, Robinette ML, 2017. SMAD4 impedes the conversion of NK cells into ILC1-like cells by curtailing non-canonical TGF-beta signaling. Nat. Immunol 18:995–1003 28759002 10.1038/ni.3809 PMC5712491

[200] Gao Y, Souza-Fonseca-Guimaraes F, Bald T, Ng SS, Young A, 2017. Tumor immunoevasion by the conversion of effector NK cells into type 1 innate lymphoid cells. Nat. Immunol 18:1004–15 28759001 10.1038/ni.3800

[201] Nixon BG, Chou C, Krishna C, Dadi S, Michel AO, 2022. Cytotoxic granzyme C-expressing ILC1s contribute to antitumor immunity and neonatal autoimmunity. Sci. Immunol 7:eabi8642 10.1126/sciimmunol.abi8642 PMC9233921 35394814

[202] Hawke LG, Mitchell BZ, Ormiston ML. 2020. TGF-beta and IL-15 synergize through MAPK pathways to drive the conversion of human NK cells to an innate lymphoid cell 1-like phenotype. J. Immunol 204:3171–81 32332109 10.4049/jimmunol.1900866

[203] Jowett GM, Norman MDA, Yu TTL, Rosell Arevalo P, Hoogland D, 2021. ILC1 drive intestinal epithelial and matrix remodelling. Nat. Mater 20:250–59 32895507 10.1038/s41563-020-0783-8 PMC7611574

[204] Wang L, Tang J, Yang X, Zanvit P, Cui K, 2020. TGF-beta induces ST2 and programs ILC2 development. Nat. Commun 11:35 31911623 10.1038/s41467-019-13734-w PMC6946674

[205] Zhang J, Qiu J, Zhou W, Cao J, Hu X, 2022. Neuropilin-1 mediates lung tissue-specific control of ILC2 function in type 2 immunity. Nat. Immunol 23:237–50 35075279 10.1038/s41590-021-01097-8

[206] Laurent P, Allard B, Manicki P, Jolivel V, Levionnois E, 2021. TGFβ promotes low IL10-producing ILC2 with profibrotic ability involved in skin fibrosis in systemic sclerosis. Ann. Rheum. Dis 80:1594–603 34285051 10.1136/annrheumdis-2020-219748 PMC8600612

[207] Bernink JH, Ohne Y, Teunissen MBM, Wang J, Wu J, 2019. c-Kit-positive ILC2s exhibit an ILC3-like signature that may contribute to IL-17-mediated pathologies. Nat. Immunol 20:992–1003 31263279 10.1038/s41590-019-0423-0

[208] Chea S, Perchet T, Petit M, Verrier T, Guy-Grand D, 2016. Notch signaling in group 3 innate lymphoid cells modulates their plasticity. Sci. Signal 9:ra45 10.1126/scisignal.aaf2223 27141929

[209] Viant C, Rankin LC, Girard-Madoux MJ, Seillet C, Shi W, 2016. Transforming growth factor-beta and Notch ligands act as opposing environmental cues in regulating the plasticity of type 3 innate lymphoid cells. Sci. Signal 9:ra46 10.1126/scisignal.aaf2176 27141930

[210] Wang S, Xia P, Chen Y, Qu Y, Xiong Z, 2017. Regulatory innate lymphoid cells control innate intestinal inflammation. Cell 171:201–16.e18 28844693 10.1016/j.cell.2017.07.027

