# Supplementary material for: TGF-β Regulation of T Cells
Source: Annu Rev Immunol. Author manuscript; Available in PMC 2025 Sep 22. (PMC12453633; doi:10.1146/annurev-immunol-101921-045939)
Supplement: 1 [file NIHMS2026162-supplement-1.pdf]

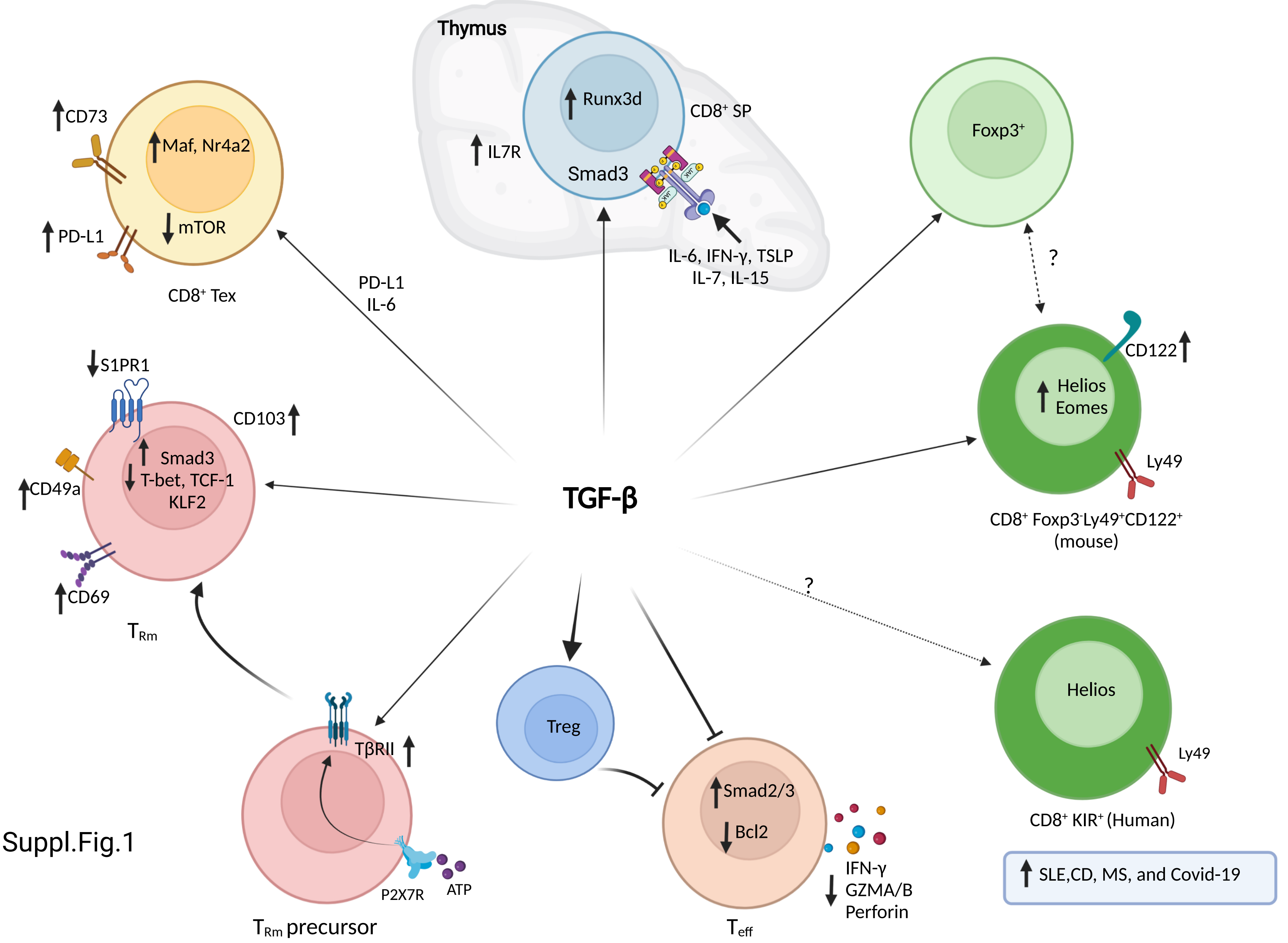

Thymus
Runx3d
CD8+ SP
IL7R
Smad3
IL-6, IFN-γ, TSLP
IL-7, IL-15
CD73
Maf, Nr4a2
PD-L1
mTOR
CD8+ Tex
PD-L1
IL-6
Foxp3+
?
CD122
Helios
Eomes
Ly49
CD8+ Foxp3-Ly49+CD122+
(mouse)
S1PR1
CD103
Smad3
T-bet, TCF-1
KLF2
CD49a
CD69
TRm
TGF-β
?
Helios
Ly49
CD8+ KIR+ (Human)
SLE,CD, MS, and Covid-19
TβRII
Treg
Smad2/3
Bcl2
IFN-γ
GZMA/B
Perforin
P2X7R
ATP
TRm precursor
Teff

Suppl.Fig.1

Supplementary Fig.1. TGF-β regulation of CD8 T cells.

Thymic CD8$^+$SP. TGF-β plays a critical role in the development of CD8$^+$ SP thymocytes. A combination of signals by IL-7 and IL-15 through common gamma-chain cytokine receptor, IL-6, IFN-γ, and TSLP through Jak-Stat, and TGF-β through Smad3 together induces the expression of the lineage-specifying transcription factor runt-related transcription factor 3d (Runx3d) and completes the generation of CD8$^+$SP thymocytes. In addition, TGF-β upregulates IL-7R expression in CD8$^+$SP.

T_{eff}. TGF-β suppresses the function of T_{eff} by inhibiting IFN-γ, Granzyme A and B, and perforin, and by promoting cell death through downregulating BCL2, in a Smad2/3 dependent manner. TGF-β may also indirectly suppress T_{eff} function by inducing Tregs.

T_{Rm}. TGF-β induces CD103, and upregulates CD49a and CD69 expression in T_{Rm} to promotes their differentiation and tissue migration. TGF-β downregulates S1PR1 expression to prevent T_{Rm} from egressing. TGF-β induces CD103 through the Smad3 binding to the *Itgae* gene, or also indirectly upregulates CD103 expression by suppressing T-bet and TCF-1 expression, as both inhibit Smad3-mediated CD103 gene transcription. TGF-β inhibits S1PR1 expression by suppressing KLF2, as KLF2 promotes *Sipr1* gene transcription. In addition, extracellular ATP senses the purinergic receptor P2RX7 on CD8$^+$ T cells to help them regain TGF-β receptors to restore their sensitivity to TGF-β, which promotes T_{Rm} precursors toward CD103$^+$ CD8$^+$ $T_{Rm.}$

T_{ex}. TGF-β may promote CD8$^+$ exhaustion during the chronic infection and cancer. This can be accomplished by upregulating the expression of PD-1 and CD73 in T_{ex}. T_{ex} express higher amounts of Nr4a2 and Maf transcription factors than do naïve CD8 T cells and T_{eff}, and Maf is upregulated by TGF-β and IL-6. TGF-β may also promote cancer exosomal PD-L1 secretion to facilitate T_{ex} formation.

CD8$^+$ Tregs. A unique population of CD8$^+$ T cells expression CD122$^+$ly49$^+$ Helios$^+$ Foxp3$^-$ have been identified as CD8$^+$ Tregs in mice. It is suggested that TGF-β and Eomes are required for their generation. The counterpart of CD8$^+$ Tregs in humans has also been identified as CD8$^+$ Ly49$^+$ KIR$^+$ Helios$^+$ cells, and they are increased in patients with SLE, colitis , MS and COVID-19. The role of TGF-β in human CD8$^+$ Tregs remains unknown. In addition, TGF-β induces Foxp3 in naïve CD8$^+$ T cells, and these CD8$^+$Foxp3$^+$ Tregs shown equal suppressive function to CD4$^+$Tregs *in vitro.* However, CD8$^+$Foxp3$^+$ T cells are hardly detectable in vivo in mice at the steady state, and the underlying mechanism remains unknown. Dotted lines indicate no experimental evidence available.

Downloaded from www.annualreviews.org. Guest (guest) IP: 156.40.252.5 On: Wed, 11 Jun 2025 16:36:55

Supplemental Material: Annu. Rev. Immunol. 2023. 41:483-512
https://doi.org/10.1146/annurev-immunol-101921-045939
TGF-β Regulation of T Cells
Chen
