# Supplementary material for: TGF-β Regulation of T Cells
Source: Annu Rev Immunol. Author manuscript; Available in PMC 2025 Sep 22. (PMC12453633; doi:10.1146/annurev-immunol-101921-045939)
Supplement: 2 [file NIHMS2026162-supplement-2.pdf]

Supplemental Material: Annu. Rev. Immunol. 2023. 41:483-512
https://doi.org/10.1146/annurev-immunol-101921-045939

TGF-β regulation of T cells

WanJun Chen

Downloaded from www.annualreviews.org. Guest (guest) IP: 156.40.252.5 On: Wed, 11 Jun 2025 16:36:40

Supplementary Text (1-6):

Supplementary Text 1: TGF-β in thymocyte development

Early evidence suggests that TGF-β inhibits $CD3^{-}CD4^{-}CD8^{-}$ triple-negative (TN) thymocyte proliferation and differentiation based on murine fetal thymus organ culture (FTOC) and human thymocyte culture (1, 2). Specifically, TGF-β inhibits the differentiation from $CD44^{+}CD25^{-}$ (TN1) to $CD44^{-}CD25^{+}$ (TN2), and consequently blocks the differentiation to double-positive (DP) thymocytes and then to $CD4^{+}$ SP thymocytes, but intriguingly not to $CD8^{+}$ SP thymocytes (2). However, others have reported that TGF-β derived from thymic cortical epithelial cells suppresses $CD4^{-}CD8^{low}$ differentiation to $CD4^{+}CD8^{+}$ DP thymocytes rather than the transition from $CD4^{-}CD8^{-}$ TN to $CD4^{+}CD8^{low}$ (3). Despite the different reports, it seems that TGF-β plays a regulatory role in the development of TN to DP thymocytes. However, with the transgenic mice in that TGF-β signaling is depleted specific in thymocytes from the early stage of DP thymocytes, one study suggested that TGF-β signals promote negative selection *in vivo* (4). However, other studies show that TGF-β signaling prevents apoptosis of thymocytes (5, 6). Thus, more investigation is warranted to resolve these discrepancies.

Supplementary Text 2: Implications of TGF-β regulation of T cell quiescence and activation

The finding that TGF-β signaling acts as a major intrinsic negative force to regulate quiescence and activation in naïve $CD4^{+}$ T cells has important implications for our understanding of immune tolerance and immune responses, as well as the development and pathogenesis of autoimmunity, cancer, and infectious disease. T cells show no reduction and, very likely, higher TβRI expression when stimulated with weak or low-dose TCR stimuli, but in comparison with naïve T cells, they exhibit a rapid and profound reduction of TβRI when activated with a strong or high dose of TCR stimuli (7). This finding may provide insight into the general lack of autoimmunity in healthy individuals, despite the existence of self-reactive T cells that regularly encounter self-antigens; this is because T cells that are normally present in the body recognize self-antigens with low affinity. "Tickling" by the low-affinity self-antigens is not sufficient to induce TβRI downregulation and even upregulates TβRI expression, and thus allows for ongoing TGF-β signaling. In cancer, the poor immunogenicity of tumor antigens may impede anti-tumor immune responses, since weak antigen stimulation does not induce TβRI downregulation. These data may also explain the phenomenon that weak TCR stimulation favors the generation of Treg cells (8, 9), as TGF-β signaling in T cells is essential for Treg cell generation (10, 11). On the other hand, T cell quiescence can be broken and T cell activation and proliferation allowed when the T cell encounters pathogens and foreign antigens that normally are strong TCR stimuli that can suppress TβRI. These findings may also provide an explanation as to why activated T cells are more resistant to suppression by Treg cells compared to naïve T cells (12, 13), as TβRI downregulation in activated T cells may contribute to their resistance to Treg suppression.

Downloaded from www.annualreviews.org. Guest (guest) IP: 156.40.252.5 On: Wed, 11 Jun 2025 16:36:40

TGF-β preservation and even upregulation of TCR-mediated TβRI downregulation in T cells may be critical in preventing T cells from abnormally activating in response to TCR stimulation by self-antigens, thus avoiding autoimmunity. This may also account for insufficient anti-tumor T cell responses in the tumor microenvironment where TGF-β is produced in large amounts and activated by different means, despite the presence of tumor antigen-specific T cells within tumors. This notion is supported by studies that show anti-tumor immunity and tumor clearance after reduction of TGF-β signaling in T cells (14, 15).

The finding that IL-6 abolishes the positive effect of TGF-β on TβRI expression in T cells provides insights into the mechanisms at play in anti-infection immunity and the development of autoimmunity. It is possible that IL-6 secreted by innate immune cells in response to bacterial or viral pathogens suppresses upregulation of TβRI by TGF-β and therefore reduces the threshold of TCR activation. Furthermore, this may help explain why autoimmunity can occur in individuals following infection: IL-6 secreted by innate immune cells in response to infection may lower the ability of TGF-β to suppress self-reactive T cells by reducing TβRI expression on T cells and may thus facilitate the differentiation of autoimmune pro-inflammatory T cells (7). Furthermore, the findings also raise the possibility that IL-6 facilitates Th17 cell generation by restraining TβRI expression to reduce TGF-b signaling, in addition to IL-6 upregulation of the IL-23 receptor (16). In sum, although many questions still remain unanswered and the exact molecular mechanisms await elucidation, these findings together indicate that TCR-mediated regulation of TGFβ-TβRI signaling acts as a crucial criterion to determine T cell quiescence and activation.

Supplementary Text 3. TGF-β in Th1 cells

Early work from three decades ago showed that TGF-β inhibits Th1 cell differentiation *in vitro* by suppressing IFN-γ production (17). This was consequently confirmed by a series of *in vivo* investigations that involved manipulating TGF-β signaling in mice(18-25). One of the most significant changes in the T cells is the drastically increased production of IFN-γ in $CD4^+$ and $CD8^+$ T cells in these knockout mice. This occurs in mice even in the steady state. The activation and expansion of Th1 cells and their increased IFN-γ production contribute to the autoimmune inflammation observed in these transgenic mice. On the other hand, the release of the TGF-β brake on IFN-γ production plays important roles in anti-tumor immunity and in immune defense against infections. For example, transgenic mice engineered to express dominant-negative TGF-β receptor II in T cells (dnTβRII) show increased Th1 cell function and decreased the tumor growth(25). TGF-β-mediated suppression in T cells is responsible for the failure of BALB/c mice to develop a Th1 response against parasitic *Leishmania major* infection(26).

Mechanistically, TGF-β may directly inhibit the expression of T-bet (26), the master transcription factor of Th1 differentiation, although it has also been reported that TGF-β can suppress the expression of IL-12 receptor II in differentiating T cells to block Th1 differentiation(27). It has been suggested that TGF-β suppression of T-bet and IFN-γ expression is through a Smad3-independent, but MAP kinase MEK/ERK dependent pathway (28), but the functions of the Smad3-dependent mechanisms need to be investigated. Of note, there are published studies showing that TGF-β could paradoxically promote both Th1 differentiation and IFN-γ production in murine and human $CD4^+$ T cells in culture(29, 30). However, the exact mechanisms underlying this discrepancy of TGF-β in Th1 cells in *in vitro* cultures remain to be elucidated.

Supplementary Text 4. TGF-β in Th2 cells.

Th2 cells are differentiated from naïve $CD4^+$ T cells upon TCR stimulation in the presence of IL-4. They play a primary role in the immune defense against parasite infections and are involved in the pathogenesis of allergy and asthma by producing signature cytokines IL-4, IL-5, and IL-13 (31). TGF-β suppresses the differentiation and function of Th2 cells *in vitro* and *in vivo* (17, 32). dnTbRII mice exhibit spontaneously Th2 cells in addition to Th1 cells, and the suppression of Th2 differentiation is mediated by TGF-β inhibition of GATA3(25), the master transcription factor for Th2 cells (31, 33). In addition, TGF-β may also indirectly suppress Th2 cells by inducing Tregs (10, 34). Interestingly, helminth secretions of TGF-β-mimic protein induce Foxp3 $^+$ Treg generation through the TGF-β signaling pathway, and these Tregs suppresses Th2 cell proliferation *in vitro* and allergic airway inflammation *in vivo* (35). In addition to the well-defined suppressive effects of TGF-β on Th2 cell differentiation, exposure to high doses of TGF-β in Th2 cells is also able to further suppress GATA3 and IL-4 production from Th2 cells and convert these Th2 cells towards Th9 cells by producing IL-9 (36) (see section 4.2.4. TGF-β in Th9 cells). A recent study has shown that TGF-β may preferably suppress IL-4-producing Th2 cells rather than Th1 cells and $CD8^+$ T cells in tumor models in mice (37). The authors showed that depletion of TβRII in $CD4^+$ T cells, but not in $CD8^+$ T cells, halted cancer progression as a result of tissue healing and remodeling of the blood vasculature, causing cancer cell hypoxia and death in distant avascular regions. They suggest that the host-directed protective response to cancer is dependent on the Th2 cytokine IL-4, but not the Th1 cytokine IFN-γ. Although this observation is interesting, it contrasts with the well-recognized evidence that TGF-β mediated suppression of anti-tumor activity significantly runs through inhibiting Th1 cells and $CD8^+$ T cells (38). Thus, more investigations are needed to resolve this discrepancy.

Supplementary Text 5. TGF-β in Tfh and Tfr

T follicular helper (Tfh) cells play a key role in generating the antigen-specific antibody response by helping B cells (39) and this is largely through the formation of a germinal center (GC) (40). Tfh cells express CXCR5 and induced costimulatory receptor (ICOS), produce IL-21 and are driven to differentiate through the transcription factor BCL-6. Early studies showed that TGF-β signaling is dispensable for Tfh differentiation driven by IL-21 *in vitro* and *in vivo*, as blockade of TGF-β with anti-TGF-β antibodies in a KLH-immunized mice fails to affect CXCR5 expression in $CD4^+$ T cells (41). Moreover, it has even been reported that TGF-β can suppress Tfh cell expansion by inducing $CD8^+CD44^{hi}$ $CD122^+Ly49^+$ regulatory T cells that drive Tfh cell death (42). However, other studies have reported that TGF-β signaling to $CD4^+$ T cells seems to be important for the formation of influenza-specific Tfh cells, GC reactions, and development of isotype-switched, flu-specific antibody responses (43). Of interest, a recent study showed that a unique population of $Foxp3^+$Tfh cells are converted by TGF-β from $Foxp3^-$ Tfh cells(44).

TGF-β may play a role in human Tfh cell differentiation. It has been shown that TGF-β provides critical costimulatory signals for Stat3 and Stat4 to promote initial Tfh differentiation in humans, but this mechanism does not appear to be shared in murine $CD4^+$ T cells(45). TGF-β acts together with IL-12 and IL-23 to upregulate CXCR5, BCL-6, ICOS, and IL-21 as well as BATF and C-Maf, but to downregulate Blimp-1 to promote Tfh cells from $CD4^+$ T cells (45, 46). Furthermore, TGF-β represses the expression of genomic organizer SATB1 in tumor infiltrated T cells(47), through Smad2/3 protein binding to the *Satab1* promoter (48). The decrease in SATB1 results in

Downloaded from www.annualreviews.org. Guest (guest) IP: 156.40.252.5 On: Wed, 11 Jun 2025 16:36:40

upregulation of PD-1 and ICOS expression in $CD4^+$ T cells, which promote Tfh differentiation and increases the isotype-switched B cell response *in vivo* in mice.

T follicular regulatory T cells (Tfr) are a specialized $Foxp3^+$ Treg subset that can access to B cell follicles and regulate Tfh mediated B cell response and humoral immunity. Mature Tfr express $CXCR5^+$,$PD\text{-}1^+$, $BCL6^+$ and $Foxp3^+$, but are $CD25^-$ and $Blimp1^-$ (49). It is suggested that Tfr are differentiated from the tTregs, but not converted from naïve $CD4^+$ $Foxp3^-$ T cells or from $Foxp3^-$ Tfh cells. In contrast to Tfh, there is no strong evidence that TGF-β is involved in the differentiation of Tfr. However, human Tfr cells express GARP, a molecule anchoring latent TGF-β on the surface of Tregs, suggesting a potential role of TGF-β in human Tfr function (50).

Downloaded from www.annualreviews.org. Guest (guest) IP: 156.40.252.5 On: Wed, 11 Jun 2025 16:36:40

Supplementary Text 6. Reconciliation between thymic apoptosis-TGF-β-tTreg model and other models of tTreg development

We proposed a model that thymocyte apoptosis is linked to the generation of tTregs in a TGF-β dependent manner(11, 51) (**Fig.2**). This model could reconcile well with the current models of tTreg development. Firstly, the agonist/high affinity/avidity TCR-driving tTreg development model can be explained alternatively that the high affinity/avidity TCR stimulation in the thymocytes increases the number of apoptotic cells and consequently drives larger amounts of TGF-β production and/or activation and consequently promotes more tTreg development (51, 52). This model may also explain why thymic tTregs cannot be detected until day 3 after the mice are born, as the apoptotic cells in the thymus suddenly increase at neonatal day 2 in normal mice (53), and the increase in apoptotic thymocytes leads to higher levels of TGF-β (51). Moreover, this model may also reconcile with the intraclonal competition model (54), in that the limited number of tTreg precursors compete not only for the peptides, but also for transient and limited amounts of active TGF-β in the thymus. Finally, this model may also reconcile with the current two-step model (55, 56). In the two-step model it has been suggested that TCR stimulation induces thymic $CD4^+CD25^+Foxp3^–$ precursors (first step), followed by IL-2 stimulation to convert them into $Foxp3^+$ tTregs (second step), and TGF-β was suggested to not be involved, as blockade of TGF-β in the second step *in vitro* failed to affect the yield of tTregs (55). However, we have recently found that the thymic $CD4^+CD25^+Foxp3^–$ precursors express *foxp3* mRNA, and TGF-β is clearly required in the first step of the model, namely the generation of $CD4^+CD25^+Foxp3^–$ (*foxp3* $mRNA^+$) precursors from $CD4^+CD25^–Foxp3^–$ SP thymocytes (our unpublished data)

Literatures cited for Supplementary text:

1. Mossalayi MD, Mentz F, Ouaaz F, Dalloul AH, Blanc C, Debre P, Ruscetti FW. 1995. Early human thymocyte proliferation is regulated by an externally controlled autocrine transforming growth factor-beta 1 mechanism. *Blood* 85: 3594-601
2. Plum J, De Smedt M, Leclercq G, Vandekerckhove B. 1995. Influence of TGF-beta on murine thymocyte development in fetal thymus organ culture. *J Immunol* 154: 5789-98
3. Takahama Y, Letterio JJ, Suzuki H, Farr AG, Singer A. 1994. Early progression of thymocytes along the CD4/CD8 developmental pathway is regulated by a subset of thymic epithelial cells expressing transforming growth factor beta. *J Exp Med* 179: 1495-506

4. McCarron MJ, Irla M, Serge A, Soudja SM, Marie JC. 2019. Transforming Growth Factor-beta signaling in alphabeta thymocytes promotes negative selection. *Nat Commun* 10: 5690
5. Chen W, Jin W, Tian H, Sicurello P, Frank M, Orenstein JM, Wahl SM. 2001. Requirement for transforming growth factor beta1 in controlling T cell apoptosis. *J Exp Med* 194: 439-53
6. Ouyang W, Beckett O, Ma Q, Li MO. 2010. Transforming growth factor-beta signaling curbs thymic negative selection promoting regulatory T cell development. *Immunity* 32: 642-53
7. Tu E, Chia CPZ, Chen W, Zhang D, Park SA, Jin W, Wang D, Alegre ML, Zhang YE, Sun L, Chen W. 2018. T Cell Receptor-Regulated TGF-beta Type I Receptor Expression Determines T Cell Quiescence and Activation. *Immunity* 48: 745-59 e6
8. Gottschalk RA, Corse E, Allison JP. 2010. TCR ligand density and affinity determine peripheral induction of Foxp3 in vivo. *J Exp Med* 207: 1701-11
9. Kretschmer K, Apostolou I, Hawiger D, Khazaie K, Nussenzweig MC, von Boehmer H. 2005. Inducing and expanding regulatory T cell populations by foreign antigen. *Nat Immunol* 6: 1219-27
10. Chen W, Jin W, Hardegen N, Lei KJ, Li L, Marinos N, McGrady G, Wahl SM. 2003. Conversion of peripheral CD4+CD25- naive T cells to CD4+CD25+ regulatory T cells by TGF-beta induction of transcription factor Foxp3. *J Exp Med* 198: 1875-86
11. Chen W, Konkel JE. 2015. Development of thymic Foxp3(+) regulatory T cells: TGF-beta matters. *Eur J Immunol* 45: 958-65
12. Korn T, Reddy J, Gao W, Bettelli E, Awasthi A, Petersen TR, Backstrom BT, Sobel RA, Wucherpfennig KW, Strom TB, Oukka M, Kuchroo VK. 2007. Myelin-specific regulatory T cells accumulate in the CNS but fail to control autoimmune inflammation. *Nat Med* 13: 423-31
13. Tu E, Bourges D, Gleeson PA, Ang DK, van Driel IR. 2013. Pathogenic T cells persist after reversal of autoimmune disease by immunosuppression with regulatory T cells. *Eur J Immunol* 43: 1286-96
14. Gorelik L, Flavell RA. 2001. Immune-mediated eradication of tumors through the blockade of transforming growth factor-beta signaling in T cells. *Nat Med* 7: 1118-22
15. Thomas DA, Massague J. 2005. TGF-beta directly targets cytotoxic T cell functions during tumor evasion of immune surveillance. *Cancer Cell* 8: 369-80
16. Zhou L, Lopes JE, Chong MM, Ivanov, II, Min R, Victora GD, Shen Y, Du J, Rubtsov YP, Rudensky AY, Ziegler SF, Littman DR. 2008. TGF-beta-induced Foxp3 inhibits T(H)17 cell differentiation by antagonizing RORgammat function. *Nature* 453: 236-40
17. Mosmann TR, Coffman RL. 1989. TH1 and TH2 cells: different patterns of lymphokine secretion lead to different functional properties. *Annu Rev Immunol* 7: 145-73
18. Kulkarni AB, Huh CG, Becker D, Geiser A, Lyght M, Flanders KC, Roberts AB, Sporn MB, Ward JM, Karlsson S. 1993. Transforming growth factor beta 1 null mutation in mice causes excessive inflammatory response and early death. *Proc Natl Acad Sci U S A* 90: 770-4

Downloaded from www.annualreviews.org. Guest (guest) IP: 156.40.252.5 On: Wed, 11 Jun 2025 16:36:40

19. Li MO, Sanjabi S, Flavell RA. 2006. Transforming growth factor-beta controls development, homeostasis, and tolerance of T cells by regulatory T cell-dependent and -independent mechanisms. *Immunity* 25: 455-71
20. Liu Y, Zhang P, Li J, Kulkarni AB, Perruche S, Chen W. 2008. A critical function for TGF-beta signaling in the development of natural CD4+CD25+Foxp3+ regulatory T cells. *Nat Immunol* 9: 632-40
21. Marie JC, Liggitt D, Rudensky AY. 2006. Cellular mechanisms of fatal early-onset autoimmunity in mice with the T cell-specific targeting of transforming growth factor-beta receptor. *Immunity* 25: 441-54
22. Shull MM, Ormsby I, Kier AB, Pawlowski S, Diebold RJ, Yin M, Allen R, Sidman C, Proetzel G, Calvin D, et al. 1992. Targeted disruption of the mouse transforming growth factor-beta 1 gene results in multifocal inflammatory disease. *Nature* 359: 693-9
23. Takimoto T, Wakabayashi Y, Sekiya T, Inoue N, Morita R, Ichiyama K, Takahashi R, Asakawa M, Muto G, Mori T, Hasegawa E, Saika S, Hara T, Nomura M, Yoshimura A. 2010. Smad2 and Smad3 are redundantly essential for the TGF-beta-mediated regulation of regulatory T plasticity and Th1 development. *J Immunol* 185: 842-55
24. Travis MA, Reizis B, Melton AC, Masteller E, Tang Q, Proctor JM, Wang Y, Bernstein X, Huang X, Reichardt LF, Bluestone JA, Sheppard D. 2007. Loss of integrin alpha(v)beta8 on dendritic cells causes autoimmunity and colitis in mice. *Nature* 449: 361-5
25. Gorelik L, Flavell RA. 2002. Transforming growth factor-beta in T-cell biology. *Nat Rev Immunol* 2: 46-53
26. Gorelik L, Constant S, Flavell RA. 2002. Mechanism of transforming growth factor beta-induced inhibition of T helper type 1 differentiation. *J Exp Med* 195: 1499-505
27. Gorham JD, Guler ML, Fenoglio D, Gubler U, Murphy KM. 1998. Low dose TGF-beta attenuates IL-12 responsiveness in murine Th cells. *J Immunol* 161: 1664-70
28. Park IK, Letterio JJ, Gorham JD. 2007. TGF-beta 1 inhibition of IFN-gamma-induced signaling and Th1 gene expression in CD4+ T cells is Smad3 independent but MAP kinase dependent. *Mol Immunol* 44: 3283-90
29. Cerwenka A, Bevec D, Majdic O, Knapp W, Holter W. 1994. TGF-beta 1 is a potent inducer of human effector T cells. *J Immunol* 153: 4367-77
30. Smeltz RB, Chen J, Shevach EM. 2005. Transforming growth factor-beta1 enhances the interferon-gamma-dependent, interleukin-12-independent pathway of T helper 1 cell differentiation. *Immunology* 114: 484-92
31. Zhu J, Paul WE. 2010. Peripheral CD4+ T-cell differentiation regulated by networks of cytokines and transcription factors. *Immunol Rev* 238: 247-62
32. Gorelik L, Flavell RA. 2000. Abrogation of TGFbeta signaling in T cells leads to spontaneous T cell differentiation and autoimmune disease. *Immunity* 12: 171-81
33. Zheng W, Flavell RA. 1997. The transcription factor GATA-3 is necessary and sufficient for Th2 cytokine gene expression in CD4 T cells. *Cell* 89: 587-96
34. Josefowicz SZ, Niec RE, Kim HY, Treuting P, Chinen T, Zheng Y, Umetsu DT, Rudensky AY. 2012. Extrathymically generated regulatory T cells control mucosal TH2 inflammation. *Nature* 482: 395-9
35. Grainger JR, Smith KA, Hewitson JP, McSorley HJ, Harcus Y, Filbey KJ, Finney CA, Greenwood EJ, Knox DP, Wilson MS, Belkaid Y, Rudensky AY, Maizels RM. 2010.

Downloaded from www.annualreviews.org. Guest (guest) IP: 156.40.252.5 On: Wed, 11 Jun 2025 16:36:40

Helminth secretions induce de novo T cell Foxp3 expression and regulatory function through the TGF-beta pathway. *J Exp Med* 207: 2331-41
36. Veldhoen M, Uyttenhove C, van Snick J, Helmby H, Westendorf A, Buer J, Martin B, Wilhelm C, Stockinger B. 2008. Transforming growth factor-beta 'reprograms' the differentiation of T helper 2 cells and promotes an interleukin 9-producing subset. *Nat Immunol* 9: 1341-6
37. Liu M, Kuo F, Capistrano KJ, Kang D, Nixon BG, Shi W, Chou C, Do MH, Stamatiades EG, Gao S, Li S, Chen Y, Hsieh JJ, Hakimi AA, Taniuchi I, Chan TA, Li MO. 2020. TGF-beta suppresses type 2 immunity to cancer. *Nature* 587: 115-20
38. Flavell RA, Sanjabi S, Wrzesinski SH, Licona-Limon P. 2010. The polarization of immune cells in the tumour environment by TGFbeta. *Nat Rev Immunol* 10: 554-67
39. Crotty S. 2011. Follicular helper CD4 T cells (TFH). *Annu Rev Immunol* 29: 621-63
40. Victora GD, Nussenzweig MC. 2012. Germinal centers. *Annu Rev Immunol* 30: 429-57
41. Nurieva RI, Chung Y, Hwang D, Yang XO, Kang HS, Ma L, Wang YH, Watowich SS, Jetten AM, Tian Q, Dong C. 2008. Generation of T follicular helper cells is mediated by interleukin-21 but independent of T helper 1, 2, or 17 cell lineages. *Immunity* 29: 138-49
42. McCarron MJ, Marie JC. 2014. TGF-beta prevents T follicular helper cell accumulation and B cell autoreactivity. *J Clin Invest* 124: 4375-86
43. Marshall HD, Ray JP, Laidlaw BJ, Zhang N, Gawande D, Staron MM, Craft J, Kaech SM. 2015. The transforming growth factor beta signaling pathway is critical for the formation of CD4 T follicular helper cells and isotype-switched antibody responses in the lung mucosa. *Elife* 4: e04851
44. Jacobsen JT, Hu W, TB RC, Solem S, Galante A, Lin Z, Allon SJ, Mesin L, Bilate AM, Schiepers A, Shalek AK, Rudensky AY, Victora GD. 2021. Expression of Foxp3 by T follicular helper cells in end-stage germinal centers. *Science* 373
45. Schmitt N, Liu Y, Bentebibel SE, Munagala I, Bourdery L, Venuprasad K, Banchereau J, Ueno H. 2014. The cytokine TGF-beta co-opts signaling via STAT3-STAT4 to promote the differentiation of human TFH cells. *Nat Immunol* 15: 856-65
46. Johnston RJ, Poholek AC, DiToro D, Yusuf I, Eto D, Barnett B, Dent AL, Craft J, Crotty S. 2009. Bcl6 and Blimp-1 are reciprocal and antagonistic regulators of T follicular helper cell differentiation. *Science* 325: 1006-10
47. Chaurio RA, Anadon CM, Lee Costich T, Payne KK, Biswas S, Harro CM, Moran C, Ortiz AC, Cortina C, Rigolizzo KE, Sprenger KB, Mine JA, Innamarato P, Mandal G, Powers JJ, Martin A, Wang Z, Mehta S, Perez BA, Li R, Robinson J, Kroeger JL, Curiel TJ, Yu X, Rodriguez PC, Conejo-Garcia JR. 2022. TGF-beta-mediated silencing of genomic organizer SATB1 promotes Tfh cell differentiation and formation of intra-tumoral tertiary lymphoid structures. *Immunity* 55: 115-28 e9
48. Stephen TL, Payne KK, Chaurio RA, Allegrezza MJ, Zhu H, Perez-Sanz J, Perales-Puchalt A, Nguyen JM, Vara-Ailor AE, Eruslanov EB, Borowsky ME, Zhang R, Laufer TM, Conejo-Garcia JR. 2017. SATB1 Expression Governs Epigenetic Repression of PD-1 in Tumor-Reactive T Cells. *Immunity* 46: 51-64
49. Wing JB, Tekguc M, Sakaguchi S. 2018. Control of Germinal Center Responses by T-Follicular Regulatory Cells. *Front Immunol* 9: 1910

Downloaded from www.annualreviews.org. Guest (guest) IP: 156.40.252.5 On: Wed, 11 Jun 2025 16:36:40

50. Sayin I, Radtke AJ, Vella LA, Jin W, Wherry EJ, Buggert M, Betts MR, Herati RS, Germain RN, Canaday DH. 2018. Spatial distribution and function of T follicular regulatory cells in human lymph nodes. *J Exp Med* 215: 1531-42
51. Konkel JE, Jin W, Abbatiello B, Grainger JR, Chen W. 2014. Thymocyte apoptosis drives the intrathymic generation of regulatory T cells. *Proc Natl Acad Sci U S A* 111: E465-73
52. Chen W, Frank ME, Jin W, Wahl SM. 2001. TGF-beta released by apoptotic T cells contributes to an immunosuppressive milieu. *Immunity* 14: 715-25
53. Surh CD, Sprent J. 1994. T-cell apoptosis detected in situ during positive and negative selection in the thymus. *Nature* 372: 100-3
54. Bautista JL, Lio CW, Lathrop SK, Forbush K, Liang Y, Luo J, Rudensky AY, Hsieh CS. 2009. Intraclonal competition limits the fate determination of regulatory T cells in the thymus. *Nat Immunol* 10: 610-7
55. Burchill MA, Yang J, Vang KB, Moon JJ, Chu HH, Lio CW, Vegoe AL, Hsieh CS, Jenkins MK, Farrar MA. 2008. Linked T cell receptor and cytokine signaling govern the development of the regulatory T cell repertoire. *Immunity* 28: 112-21
56. Lio CW, Hsieh CS. 2008. A two-step process for thymic regulatory T cell development. *Immunity* 28: 100-11

Downloaded from www.annualreviews.org. Guest (guest) IP: 156.40.252.5 On: Wed, 11 Jun 2025 16:36:40
